# Simulated radiation levels and patterns of MRI without a Faraday shielded room

**DOI:** 10.1002/mrm.30499

**Published:** 2025-03-17

**Authors:** Ehsan Kazemivalipour, Bastien Guerin, Lawrence L. Wald

**Affiliations:** ^1^ A. A. Martinos Center for Biomedical Imaging, Department of Radiology Massachusetts General Hospital Charlestown Massachusetts USA; ^2^ Harvard Medical School Boston Massachusetts USA; ^3^ Harvard‐MIT Division of Health Sciences Technology Cambridge Massachusetts USA

**Keywords:** birdcage coil, magnetic resonance imaging, radiation pattern, safety, shielded room

## Abstract

**Purpose:**

We characterize electromagnetic (EM) radiation patterns and levels in conventional MRI systems as a function of field strength and load symmetry, providing a framework for mitigation strategies allowing operation without a shielded room.

**Methods:**

We simulated the far‐field radiation pattern and fields at a 10 m radius (|E|_10m_ and |B|_10m_) for a solenoidal superconducting MRI with a body birdcage coil operated between 0.25T and 6.5T. Five load configurations probed the impact of load‐symmetry, ranging from a sphere to a body load (least‐symmetric). We also assessed simple layered EM absorbers at the bore‐ends.

**Results:**

All configurations exceeded regulatory limits for realistic transmit levels. At 1.5T, a 300 V_rms_ RF‐pulse is 2700‐fold the |E|_10m_ limit. Field strength and load symmetry strongly modulate radiation patterns and levels. The radiated power increased by more than four orders of magnitude from 0.25T to 6.5T. Spherical load radiation transitioned from a peak gain at the bore‐ends (0.25–0.5T) to a donut‐shaped pattern, suggesting current loops around the bore (1 T–1.5T), back to bore‐axis‐directed gain, suggesting propagating waves along the bore (2T–6.5T). Transition patterns were seen between these regimes; uniform radiation at 0.75T and a combined donut/bore‐directed pattern at 1.75T. Load asymmetry increased both strength and pattern asymmetry, with the body load having the highest and least symmetric radiation with the legs facilitating wave propagation at high‐fields. A simple optimized layered absorber at scanner's service‐end reduced 3T peak radiation by 11 dB.

**Conclusion:**

Radiation from unshielded scanners far exceeds regulatory limits, particularly at high‐field. Mitigation strategies must address load‐symmetry, field strength, and wave effects.

## 1 INTRODUCTION

MRI is a common imaging technique but is expensive and, therefore, suffers from poor accessibility in low‐resource countries as well as the rural areas of high‐income countries.^1^, ^2^, ^3^, ^4^, ^5^, ^6^, ^7^, ^8^, ^9^ Siting requirements constitute a significant fraction of the total cost of installing an MRI system, which negatively affects the accessibility of this essential healthcare technology. For example, the specialized construction constraints associated with siting a superconducting solenoidal‐magnet type MRI scanner for safe operation in hospital settings can double the total actual cost.^10^, ^11^ Thus, decreasing siting costs is an attractive path toward increasing MRI accessibility. The Faraday shielded room is one of the more expensive siting requirements for conventional MRI suites, costing well over $100 000 USD.^10^, ^12^

The shielded room has two purposes. Its first role is to address what we term the “receive problem”: the attenuation of external electromagnetic interference (EMI) sources to prevent image artifacts generated by their detection by the scanner's receive system.^13^ The second role is to attenuate the transmitted RF radiation produced by the scanner's transmit coil during excitation (the “transmit problem”).^14^, ^15^, ^16^, ^17^ The Faraday shield's attenuation of the stray transmit fields prevents disruption of nearby equipment such as life‐support systems and critical monitoring devices whose electromagnetic compatibility (EMC) certifies operation only up to a prescribed level of external interference.^14^, ^15^, ^16^, ^17^ The receive problem can be addressed through digital processing techniques that are available for detecting and removing artifacts. For example, external detection coils and post‐processing algorithms can eliminate identified external interference from the image.^18^, ^19^, ^20^, ^21^, ^22^, ^23^, ^24^, ^25^ The transmit problem is less well studied, and the focus of this work is to provide a detailed characterization of the problem as the starting point for mitigation strategies, such as the use of external absorbers, body‐coil geometry changes, or the placement of emitters aimed at far‐field cancellation of the radiation.

RF coils are designed to generate a well‐defined B_1_^+^‐field distribution within the imaging volume while ensuring acceptable specific absorption rates (SAR) within the body, but the radiated power is generally ignored because it is typically only a small fraction of the input power (<1% at 1.5T) and is known to be adequately absorbed by the Faraday shielding. Operation without Faraday shielding will ultimately require the radiated power levels to be mitigated, the strategies for which must be informed by the characterization and understanding of the expected patterns and levels.

Electromagnetic (EM) radiation during the transmit phase originates from the transmit coil and currents it induces in the scanner's surrounding conductive elements. These are dependent on the Tx coil type, scanner geometry, and operating frequency. The induced currents and radiation pattern are further affected by load placement and symmetry, which can impact both the induced currents and traveling wave effects in the bore. The latter can occur at high frequencies where the shorter wavelength supports traveling wave propagation within bore.^26^

In this study, we apply EM modeling of the RF field patterns emitted from a birdcage body coil inside a superconducting solenoid magnet. We extend the EM modeling beyond 10 m from isocenter to characterize the EM radiation expected from MRI systems operating without a shielded room and thus assess the severity of the transmit problem and provide a framework to assess active and passive mitigation methods. We examine the radiation patterns and levels as a function of field strength and load symmetry, comparing them to regulatory limits. The International Electrotechnical Commission (IEC) has safety standards for medical electrical equipment, including MRI scanners, which are categorized as group II, class A devices, and must adhere to IEC 60601‐1‐2.^27^ The IEC standard in turn refers to CISPR 11^28^ for regulatory control of EM radiation. These limits protect nearby equipment from excessive EM radiation and ensure operation within their radiation tolerance levels. Thus, the hundreds of supplemental devices in the hospital must be tested to withstand interference up to these levels, and relaxing the limits would incur enormous re‐testing costs even if it were largely safe.

The IEC limits vary according to the operational (Larmor) frequency and specify peak electric (|E|) or magnetic (|B|) field levels produced at a nominal distance of 3, 10, or 30 m. We consider the peak |E| and |B| fields on the 10 m radius sphere: peak‐|E|_10m_ and peak‐|B|_10m_. For frequencies below 30 MHz, the limit is expressed as the maximum magnetic field amplitude, peak‐|B|_10m_. Thus, for clinical scanners with B_0_ between 93 mT and 0.47T, the maximum allowable peak‐|B|_10m_ is 10.59 pT (18.5 dBμA/m), and for scanners between 0.47T and 0.7T, the limit is 3.35 pT (8.5 dBμA/m). For operating frequencies above 30 MHz, the peak‐|E|_10m_ is limited, with values ranging from 50 to 78 dBμV/m (0.3–7.9 mV/m) depending on the frequency.^28^ Specifically, the regulations mandate that peak‐|E|_10m_ should not exceed 50 dBμV/m (0.32 mV/m) for 1.5T scanners and 60 dBμV/m (1 mV/m) for 3T and 7T. Note that, unlike SAR, the regulations are stated in terms of instantaneous peak fields, not time‐averaged quantities. Thus, the peak‐|E|_10m_ and peak‐|B|_10m_ scale proportionally to the amplifier drive voltage applied to the birdcage ports and do not depend on the pulse duty‐cycle.

We assess the prospect of operating MRI scanners without the Faraday shield by analyzing the RF radiation patterns and E and B amplitudes at a 10 m radius from isocenter for a generic superconducting solenoid scanner geometry transmitting with a circularly‐polarized (CP) birdcage body coil. We modeled this system geometry for RF emission at 12 proton Larmor frequencies corresponding to field strengths spanning from 0.25T to 6.5T (the highest frequency at which the birdcage coil could be tuned) and compared to regulatory levels. We investigate the influence of load asymmetry on radiation patterns, considering different load types ranging from simple geometric shapes to anatomically realistic human body models.

## 2 METHODS

### 2.1 MRI EM Modeling

**FIGURE 1:**
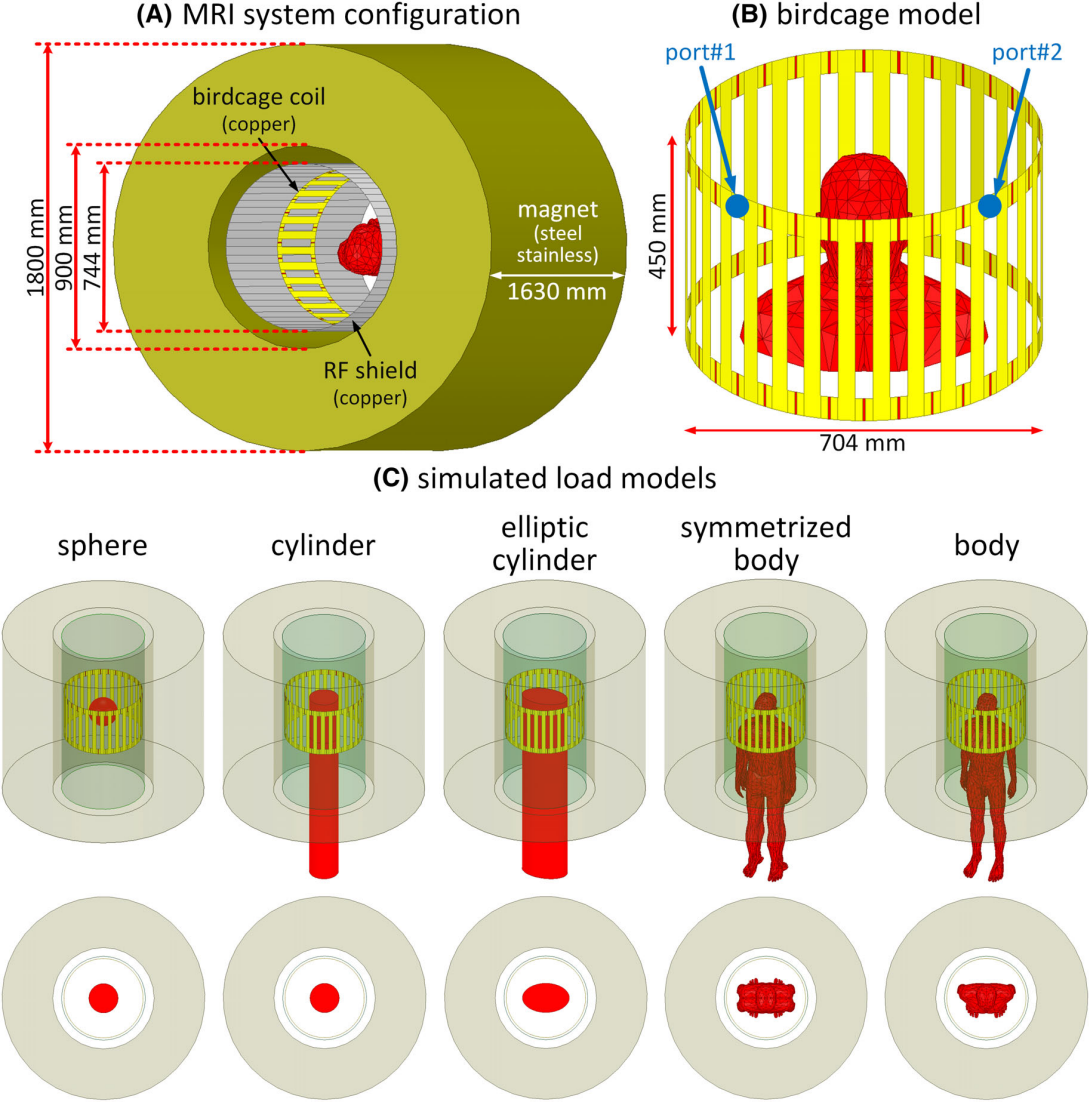
Overview of simulated MRI system (mimicking Siemens 3T Skyra scanner) without a shielded room including magnet, RF shield, 2‐port drive, 32 rung, high‐pass RF birdcage coil, and load. (A) Materials and dimensions. (B) Detail of the birdcage coil driven in the circularly polarized (CP) mode. (C) The five loads analyzed: Sphere, cylinder, elliptic cylinder, symmetrized body, and body positioned for head imaging, all filled with material mimicking average body electrical properties.

Figure 1 illustrates the simulated MRI system: a solenoidal magnet, a cylindrical RF‐shield which is typically mounted on the inside surface of the gradient coil, and a 32‐rung high‐pass birdcage coil driven in quadrature‐mode. The dimensions of the scanner (marked on the figure) correspond to those of a 70 cm diameter patient space Siemens MAGNETOM Skyra 3T superconducting magnet‐based MRI. Figure 1C shows the five investigated loads in order of increasing asymmetry: sphere, cylinder, elliptic cylinder, symmetrized body, and body. These loads were chosen to span a range of symmetry conditions, from idealized geometries to an anatomically realistic body model. This allows the determination of what aspect of the body model's asymmetry contributes the most to the asymmetric radiation patterns seen for the full‐body model. The sphere is, of course, perfectly symmetric but retains only axial symmetry when viewed in combination with the magnet and shield. The cylinder is axially symmetric but is offset along the z‐axis to mimic a patient at the head imaging position. The elliptical cylinder breaks the axial symmetry, and the symmetrized body model further reduces axial symmetry but retains mirror symmetries. Examining the symmetry of the radiated fields provides practical insights into the most important sources of the observed radiation pattern's asymmetry expected for patient imaging. All the loads were modeled as filled with uniform material with a body‐average conductivity and dielectric constant at each field strength (Table S1). The sphere and cylinder's diameter were 260 mm. The cylinder and elliptical cross‐sectional load had a length of 1835 mm. The elliptic cylinder's major axis was 416 mm with a minor axis of 260 mm. The uniform body load was derived from the outer surface of the male ANSYS body model (ANSYS Inc., Canonsburg, PA). In the case of the symmetrized body load, two ANSYS body models were placed in opposite orientations and concatenated to create a body load symmetrized in the anterior to posterior dimension. Both cylinder loads share the same height as the body load and all models were positioned to mimic a head imaging landmark.

### 2.2 Numerical EM Simulations

EM simulations were conducted using ANSYS Electronics Desktop (HFSS 2024 R2, Designer, ANSYS Inc., Canonsburg, PA). The simulation software simulates the fields inside an “open” radiation box, which is sized to be at least a quarter‐wavelength beyond the magnet/load structure. This box was assigned the radiation boundary condition, meaning the simulation assumes the waves will continue to propagate. Thus, the simulation space ranged from 21 m × 21 m × 21.6 m for the 0.25T simulations to 2.7 m × 2.7 m × 3.3 m for the 6.5T simulations. The fields on the 10 m radius sphere were reported using the ANSYS field report to extrapolate the fields from the simulation radiation box region to 10 m. The radiative “far‐field” (Fraunhofer) region is generally defined to be a distance from the antenna of a maximum overall dimension of *D* greater than *2D* ^*2*^ */λ*, *λ* being the wavelength.^29^ Note the induced currents involved in the radiation can be distributed on the birdcage shield, magnet, and even body. Considering the magnet and body and setting *D* = 2.5 m, the 10 m radius surface is in the far‐field for all the frequencies studied except at 6.5T. The accuracy of the ANSYS field report was checked by running a simulation with a radiation box large enough to include the 10 m radius sphere for the 1.5T case.

The RF coil and RF‐shield were modeled using copper and stainless‐steel materials for the magnet. All were defined as good (but not perfect) conductors with stock electrical properties with conductor boundary conditions defined as HFSS's finite conductivity boundary. The effect of adding a 4 m × 4.5 m × 3 m Faraday shield to the 1.5T system loaded with a body load was also examined with a copper Faraday shield defined in HFSS similarly to the other copper elements.

ANSYS HFSS employs an adaptive mesh scheme that progressively refines an initial mesh during iterative passes subject to constraints. For each simulation, the mesh size was constrained to be less than 1 mm on lumped‐ports, less than 4 mm on the birdcage coil, less than 10 mm within the load volume, and less than 20 mm on the RF‐shield. The mesh size constraints for the magnet and radiation box surfaces were <40 mm and <250 mm, respectively. During each adaptive pass, all scattering parameters (S‐parameters) are calculated and compared with the previous pass. If the two passes do not agree, some mesh sizes are reduced. Convergence was determined by monitoring the magnitude of the S‐parameters, and simulations were considered converged when the change between consecutive passes fell below a predefined threshold of 0.01. Ensuring convergence of the S‐parameters through this criterion guaranteed the convergence of radiated power from the MRI system.

We employed the co‐simulation approach^30^ which replaces the birdcage's capacitors with lumped‐ports and then optimizes their lumped‐element values to minimize reflections at the ports for the desired CP‐mode at the Larmor frequency. This expanded the port count to 64, including two actual birdcage input ports and 62 ports corresponding to tuning capacitors. Matching capacitors were excluded from EM simulation and instead considered within the ANSYS Electronics circuit simulator. Although co‐simulation method requires more time and computational resources for field calculations, it provides the advantage of adjusting and matching the values of the lumped‐elements for the specific frequency and load using a circuit model of the coil within a circuit simulator. We more fully describe this rapid circuit simulator in our previous studies.^31^, ^32^, ^33^, ^34^, ^35^, ^36^, ^37^, ^38^, ^39^, ^40^, ^41^, ^42^ Subsequently, the EM simulation computes the S‐parameters and associated EM‐fields for each of the 64 ports of the loaded birdcage coil at the Larmor frequency.

Following the EM simulation and circuit simulation, we imported the 64‐port S‐parameters into MATLAB, to determine the optimal tuning and matching capacitors which reduce reflections at the two drive ports to below −20 dB. This optimization was done for each field strength and each of the five load conditions. The E‐fields and radiation gain patterns were then assessed with the tuned and matched coil in CP‐mode, where both ports were fed equally with a 90‐degree phase difference. The radiation gain pattern, *G*, was computed as *G(θ, φ) = P*_out_ *(θ, φ)/P*_*in*_, where *P*_out_ represents the power radiated into a specific direction (defined by the radial and azimuthal angle *θ* and *φ*), and *P*_in_ is the total power accepted into the input divided by 4π square radians. That is, *P*_in_ is the power that would be radiated into the specific direction's unit solid angle by a lossless isotropic radiator. The peak directional gain is reported as *G*_max_. Additionally, we calculated the |E|‐field or |B|‐field pattern on the surface of a 10 m radius sphere centered on the isocenter (|E|_10m_ or |B|_10m_). The RF input amplitude was either a 1‐volt RMS total input voltage, the input voltage needed to generate a global‐SAR of 2 W/kg in the load with a 10% duty‐cycle, or the voltage required for a mean B_1_^+^ of 10μT (˜ 90° excitation for a square pulse with a *τ* ≈ 0.6 ms) in the central axial slice of the load. The peak‐|E|_10m_ or |B|_10m_ is considered depending on whether the regulatory standards specify an E‐ or B‐field limit.

### 2.3 EM Absorber

High‐dielectric EM absorbers^43^, ^44^, ^45^ with optimized dielectric constants and loss tangents have the potential to mitigate EM radiation when strategically placed in the radiation field. We examined absorbing structures placed at the end of the 3T model system's bore to absorb the emitted EM waves. The absorbers included the ANSYS HFSS Perfectly Matched Layer (PML) boundaries, or a five‐layer domed dielectric absorber at the service‐end whose conductivity and dielectric constant were optimized to reduce peak‐|E|_10m_. HFSS's PMLs are characterized by complex and anisotropic material properties designed to absorb incident EM‐fields and can be conveniently generated using the PML Wizard. In our simulations, we employed cylindrical disk‐shaped PMLs with a thickness of 797.6 mm (automatically calculated by HFSS) and a diameter of 1800 mm, matching the outer diameter of the MRI magnet. These PMLs had anisotropic relative permeability tensors: T (1, 1) = T (2, 2) = 1.64 and T (3, 3) = 0.3, with zero electrical conductivity. The dielectric and magnetic loss tangents were also anisotropic, given by T (1, 1) = T (2, 2) = 1 and T (3, 3) = −1. The five‐layer domed absorber was formed from five lossy but isotropic dielectric layers, each with a spherical curvature. The outermost layer aligns with the outer circular boundary of the MRI magnet for optimal coverage, while the innermost layer conforms to the magnet warm‐bore diameter (900 mm). The five 101.6 mm thick layers were evenly spaced and the constraints on conductivity and dielectric constant were imposed to create a gradual transition in dielectric and conductivity properties, facilitating seamless absorption of RF radiation. HFSS's Pattern Search optimizer was utilized to find the relative permittivities and conductivities that minimize peak‐|E|_10m_ at the 3T Larmor frequency.

## 3 RESULTS

**FIGURE 2:**
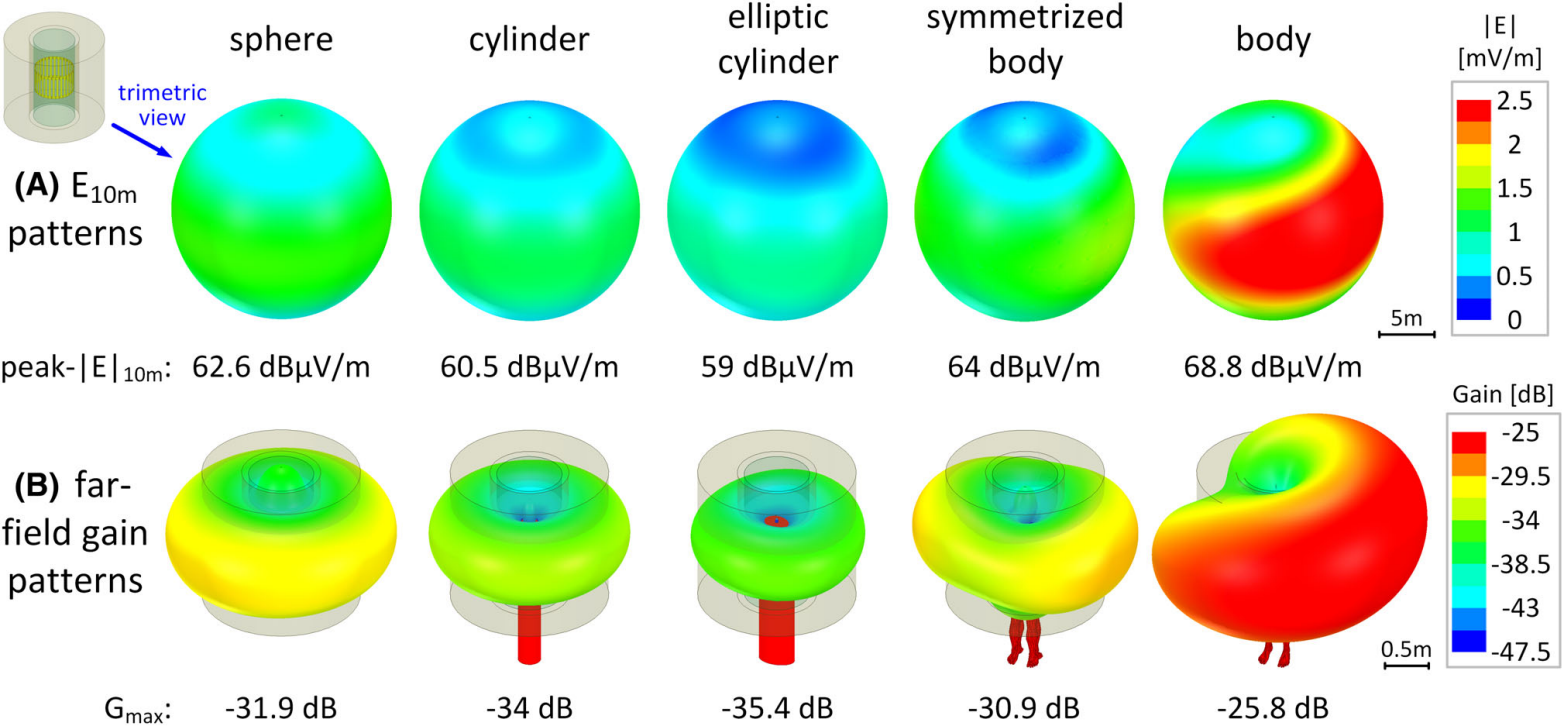
|E|_10m_ and power gain patterns (trimetric view) for an unshielded 1.5T MRI system under various loads and CP excitation mode driven with 1 V_rms_ total input (0.71 V_rms_ per port). (A) |E|_10m_ patterns and (B) far‐field gain patterns where the gain represents the directional radiated power from the scanner relative to that of an isotropic radiator with equal input power. Figure S2 shows the same data viewed from the service‐end.

The |E|_10m_ or |B|_10m_ generated by the simulated scanners significantly exceeds the regulatory limits for all field strength MRI scanners for realistic transmit levels, underscoring the challenge of replacing the Faraday shielding with other mitigation strategies to effectively contain the EM radiation within the IEC limits for a conventional superconducting scanner. Adding the 4 m × 4.5 m × 3 m copper Faraday shielding around the 1.5T system with a body load reduced the peak‐|E|_10m_ by 84 dB, bringing it below the regulatory limit (Figure S1). However, when the Faraday shielded room door is open (also shown in Figure S1), the peak‐|E|_10m_ returns to levels comparable to the unshielded case, although the 10 m field pattern is different from the unshielded case in that it is strongly peaked in the direction of the open door. Figure 2 shows the |E|_10m_ level and far‐field gain patterns for the 1.5 T system loaded with each of the 5 loads without the Faraday shielding, characterizing the degree of mitigation that will be required for shield‐less operation. Even with an input voltage of only 1 V_rms_, the peak‐|E|_10m_ for the body load exceeds the IEC regulation by 18.8 dB. A standard RF pulse with a peak of 300 V_rms_ increases the |E|_10m_ by an additional 49.5 dB and will exceed the regulatory limit by 68 dB (2600‐fold). In a practical imaging scenario where square pulses are applied in the 1.5T scanner at a 10% duty‐cycle at an amplitude that generates a global‐SAR of 2 W/kg into the body model, a 490.6 V_rms_ input is needed and the non‐shielded 1.5T MRI system would create a peak‐|E|_10m_ that is 72.6 dB higher than the regulatory limit. The symmetry of the |E|_10m_‐patterns degrades considerably as the symmetry of the load is varied, with the body load exhibiting higher and more anisotropic |E|_10m_ and gain patterns compared to symmetric loads. Notably, the body load shows a peak‐|E|_10m_ and *G*_max_ that are 6.2 dB and 6.1 dB higher than those of the sphere load. This asymmetry is largely attributed to the removal of anterior–posterior mirror symmetry in the body load, highlighting the critical role of this factor in asymmetrizing the radiation pattern. While Figure 2 presents patterns from a trimetric‐view, Figure S2 shows the same data plotted from a service‐end view.

**FIGURE 3:**
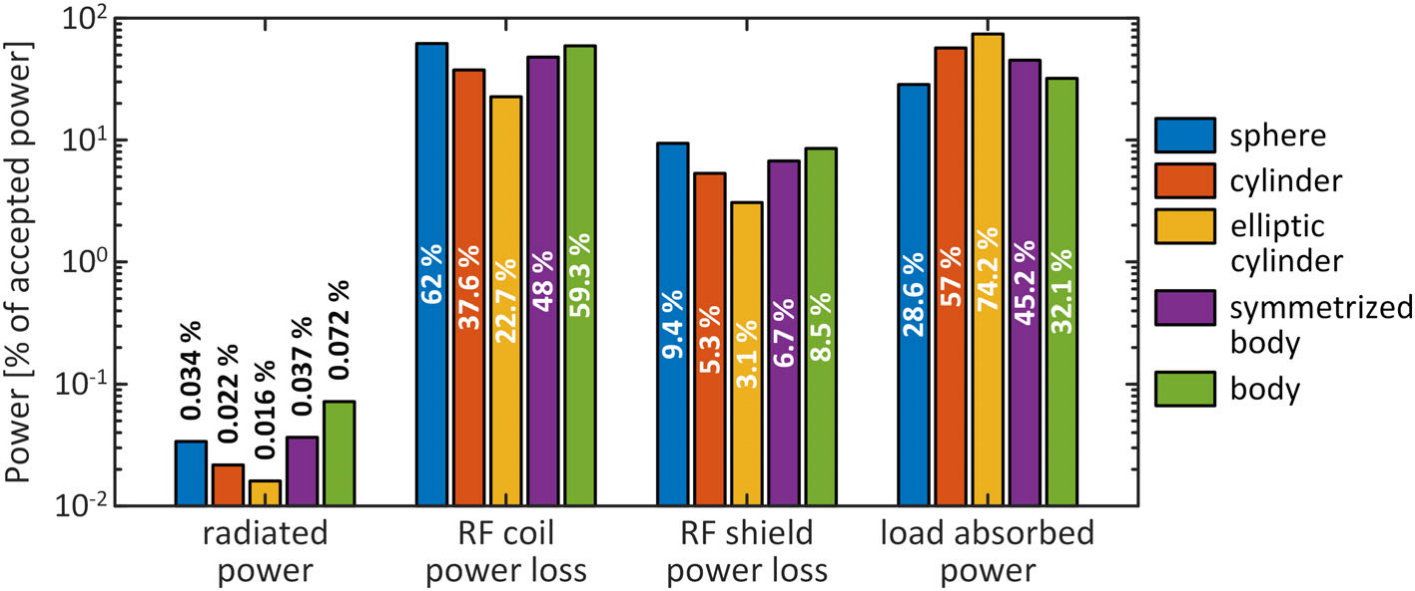
Normalized power analysis: Fraction of the accepted (forward) power which is: (1) radiated, (2) dissipated in the birdcage coil conductors, (3) dissipated in the RF‐shield, or (4) dissipated in load for various loads (coded by the color of the bar). The fractional powers are assessed for the unshielded 1.5T MRI system operating in the CP mode.

Figure 3 compares the power emitted as radiation as well as dissipated in the birdcage coil, and RF‐shield's conductors, and load as a fraction of the forward power accepted by the birdcage for the 1.5T MRI scanner with each load, in the absence of the Faraday shielding. The radiated power fraction was the highest for the body load, which was more than two‐fold that of the spherical load. The B_1_^+^‐field patterns produced in each of the 5 loads are shown in Figure S3. The spherical phantom shows the highest central B_1_^+^‐efficiency. Figure S4 shows B_1_^+^‐maps for each of the 12 field B_0_ strengths studied.

**FIGURE 4:**
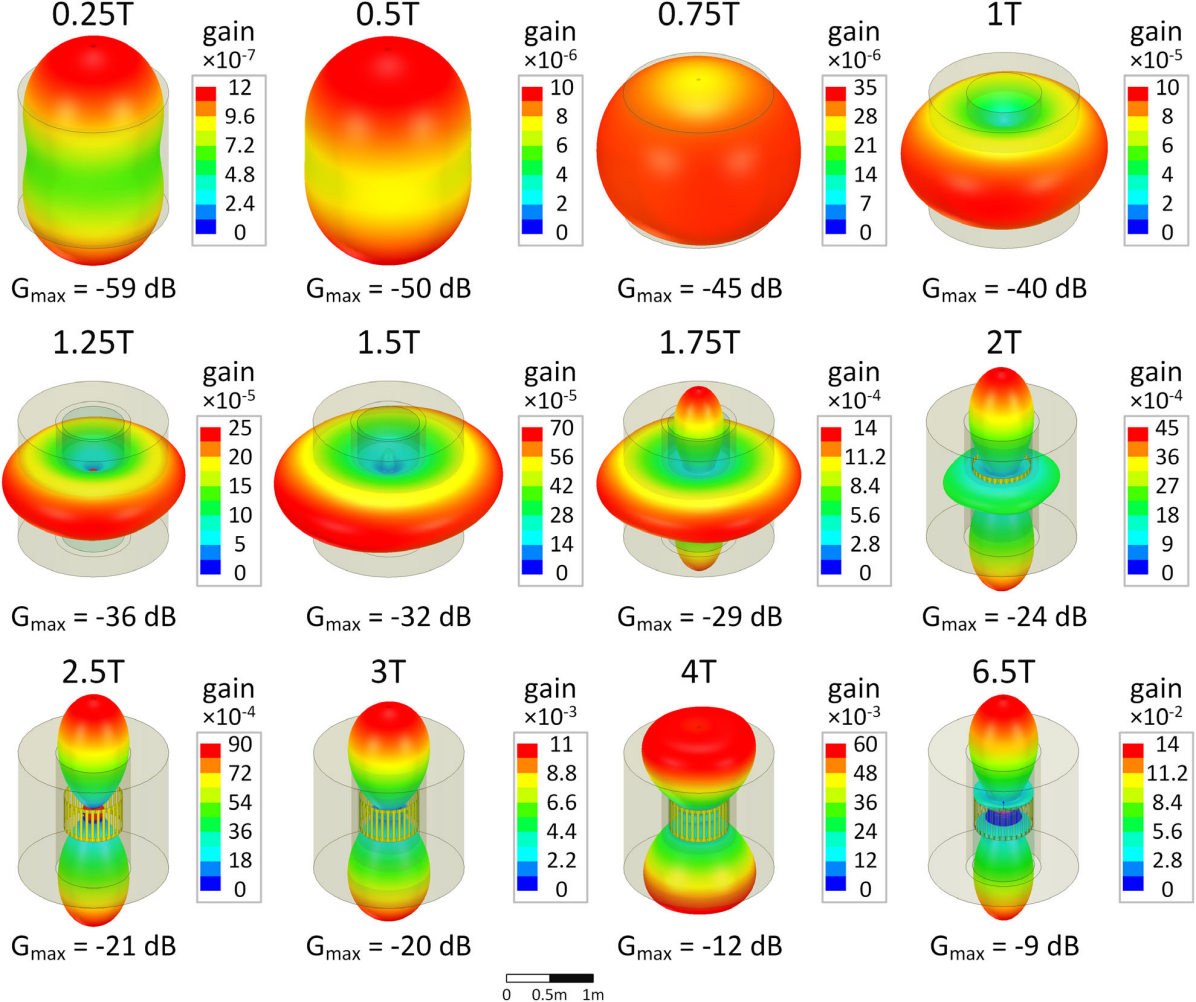
Far‐field gain patterns of unshielded MRI systems as a function of field strength. Patterns were determined using a CP‐driven birdcage coil loaded with a sphere. The gain pattern qualitatively changes from an emission pattern emanating out the bore‐ends at very low and high fields to a pattern dominated by emission radiating from the waist of the magnet at intermediate fields (1T to 1.5T). The maximum gain (*G*_*max*_) is provided for all cases.

Figure 4 depicts the far‐field gain patterns expected for each operating frequency (corresponding to B_0_ fields between 0.25T to 6.5T) using the sphere load. At least three distinct patterns are observed: (1) The “low‐field pattern” (0.25T and 0.5T) consists of a gain pattern mildly peaked at the bore‐ends. (2) The “mid‐field pattern” (1T to 1.5T) radiates in a donut‐like shape, with the maximum intensity of radiation directed perpendicular to the scanner bore. This is the expected pattern from a current circulating around the magnet or shield bore. The wavelength becomes comparable to the magnet's circumference, facilitating the formation of loop currents on the conductive surfaces, such as the RF‐shield and/or magnet cryostat. These oscillating loop currents, like a loop antenna, create strong transverse electric fields that result in donut‐shaped radiation pattern in the plane of the loop. (3) The “high‐field pattern” (2T and above), the radiation emerges from the bore‐ends. Here, the shorter wavelength allows traveling waves to propagate within the MRI bore, which acts as a cylindrical waveguide‐like structure. In this regime, first the TE_11_ mode and ultimately other traveling wave modes are supported on the cylindrical waveguide bore as determined by its cutoff frequencies.^26^ The traveling wave results in a bore‐axis‐directed radiation pattern analogous to flared waveguide antennas, where waves propagate within the structure and radiate strongly from the open ends. The 0.75T radiation pattern can be viewed as a transition from pattern 1 to 2 and the 1.75T pattern can be viewed as a transition from pattern 2 to 3. While patterns 2 and 3 are relatively easy to understand, pattern 1 is less intuitive. In this case, the wavelength is relatively long compared to the system dimensions, resulting in quasi‐static conditions and an oscillating charge of uniform phase over the structure, which is small compared to the wavelength. This would suggest that the structure would radiate more and more isotopically as the frequency is lowered. Instead, the pattern reverts to a bore‐end dominated pattern more similar to the high‐field pattern at the two lowest frequencies (0.25T and 0.5T). The 6.5T scanner generates a 50 dB higher *G*_max_ than the 0.25T scanner.

**FIGURE 5:**
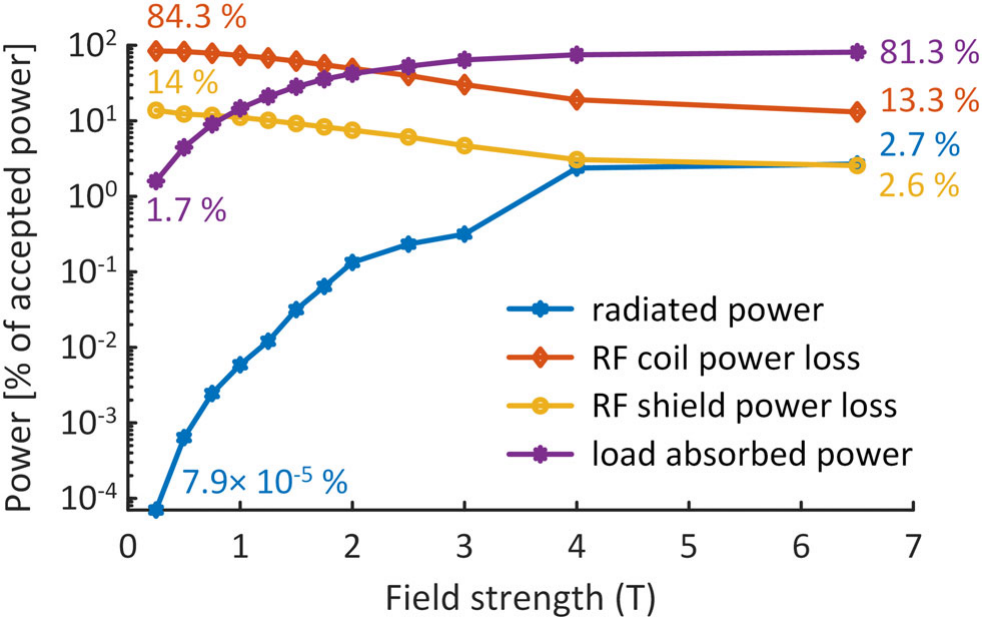
Power analysis of the unshielded MRI systems with a spherical load as a function of field strength. For each field strength, normalized radiated power, RF coil power loss, RF shield power loss, and load‐absorbed power are reported. All power values presented are normalized to the power accepted (forward power) by the CP‐driven birdcage coil. The normalized power values at 0.25T and 6.5T are provided above the respective curves for reference.

Figure 5 shows the relative power levels which are ultimately deposited in (1) the birdcage coil's conductors, (2) the RF‐shield, (3) the spherical load, and (4) radiation. These power fractions are listed as a percentage of the accepted (forward) power incident to the birdcage coil for each B_0_ field strength. The fractional power deposited in the load and emitted as radiation increases monotonically and rapidly with frequency (B_0_ field) at the expense of the relative power deposited in the coil and shield. Since the coil was well‐matched in all cases, the same relationship holds for the absolute power levels deposited in these structures.

**FIGURE 6:**
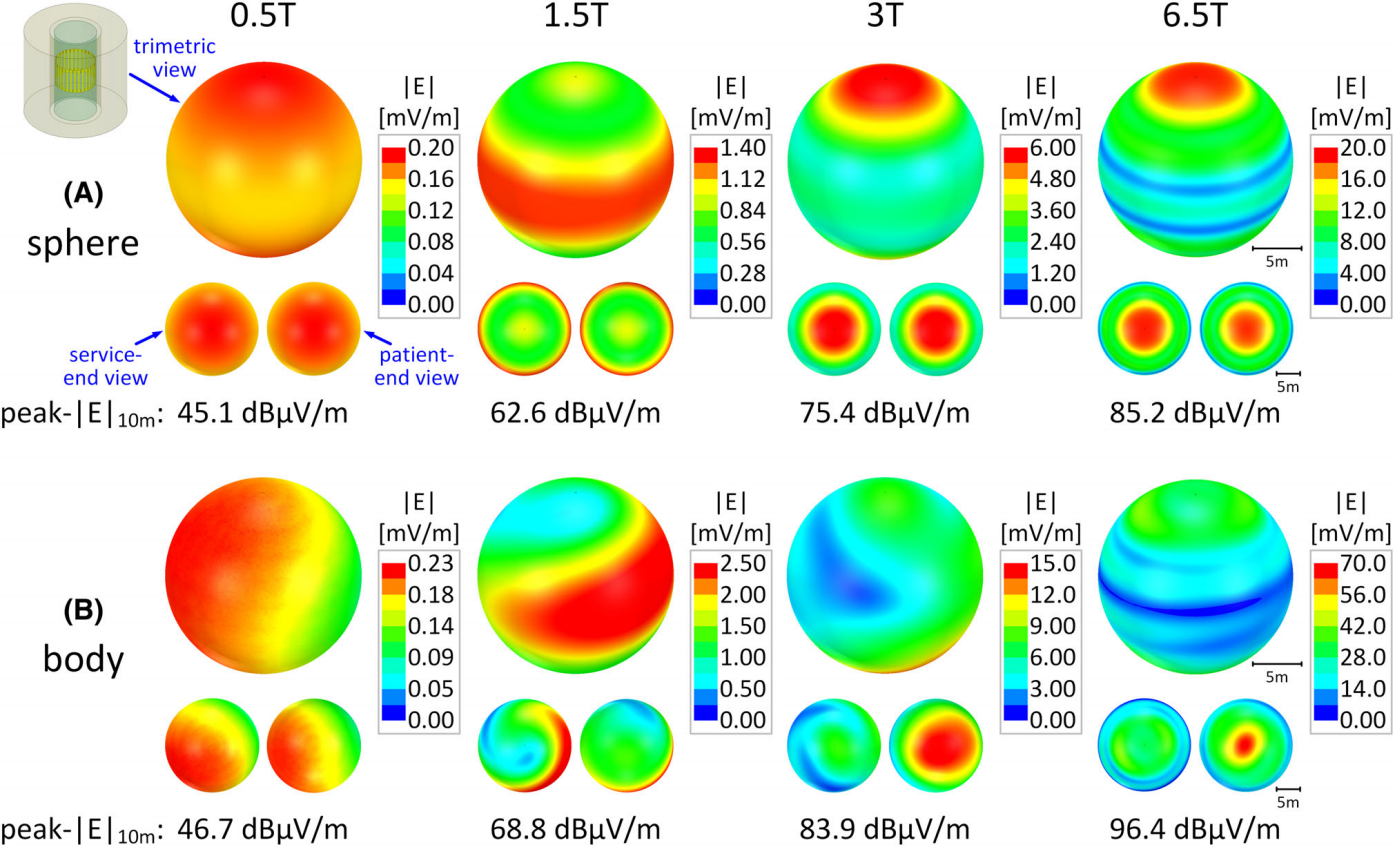
|E|_10m_ patterns for 0.5T, 1.5T, 3T, and 6.5T unshielded MRI systems operating in the CP excitation mode driven with 1 V_rms_ total input (0.71 V_rms_ per port) and loading with either (A) sphere or (B) body. |E|_10m_ patterns were shown in three different views: A side‐on view and views looking down either the service‐end or patient‐end.

**FIGURE 7:**
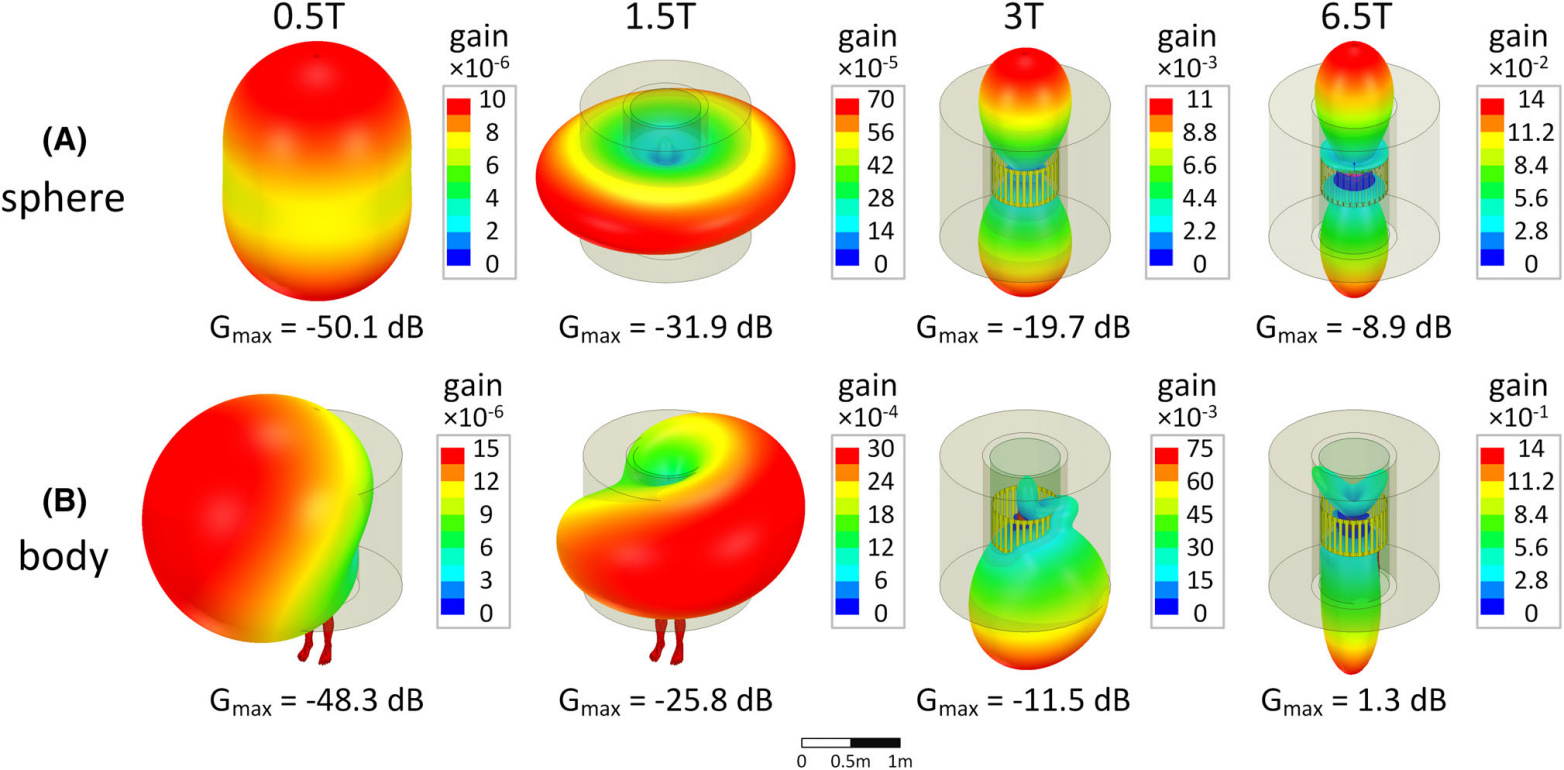
Far‐field gain patterns for 0.5T, 1.5T, 3T, and 6.5T unshielded MRI systems operating in the CP excitation mode driven with 1 V_rms_ total input (0.71 V_rms_ per port) and loading with either (A) sphere or (B) body.

Figures 6 and 7 show the |E|_10m_‐field and gain patterns, respectively, at 0.5T, 1.5T, 3T, and 6.5T for sphere and body loads, with the CP‐mode birdcage coil with 1 V_rms_ total input. The |E|_10m_ and gain patterns emphasize the influence of both RF frequency and load symmetry on MRI EM radiation. For both the sphere and body loads, the |E|_10m_ intensity and *G*_max_ increase with the field strength. For instance, the 6.5T MRI system produced 40.1 dB higher |E|_10m_ than the 0.5T system for the spherical load. For the body load, the 6.5T system generated 49.7 dB higher |E|_10m_ than the 0.5T system. The ratio of the peak‐|E|_10m_ for the body and sphere loads was 1.6 dB at 0.5T and increased to 11.2 dB at 6.5T, highlighting a stronger load dependency at higher fields. For all field strengths, the body load asymmetry introduces |E|_10m_ and gain patterns asymmetries. The gain plots of Figure 7 show that for 3T and above, most of the radiation emanates from the foot‐end of the scanner bore.

**FIGURE 8:**
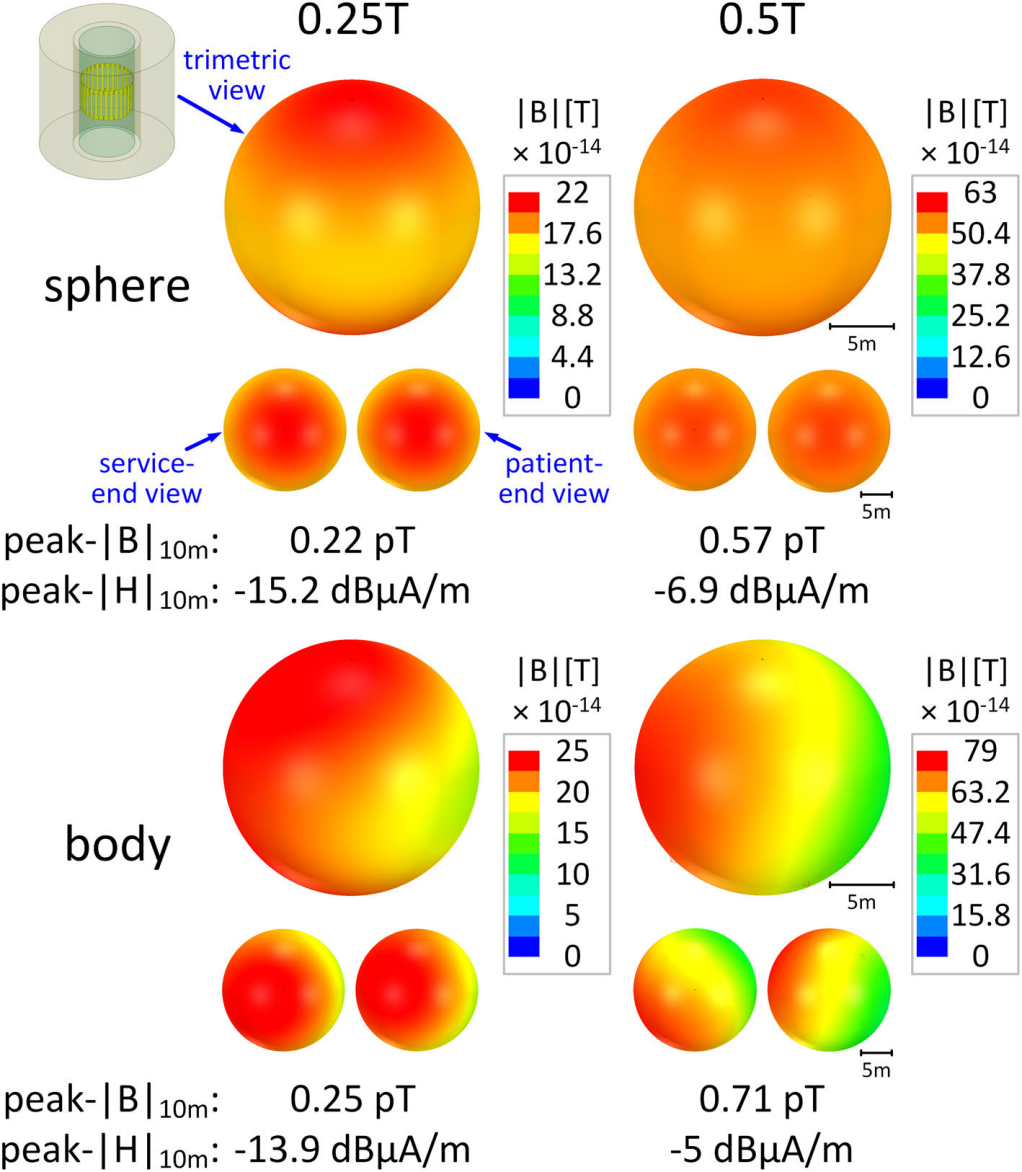
|B|_10m_ patterns for 0.25T and 0.5T unshielded MRI systems operating in the CP excitation mode driven with 1 V_rms_ total input (0.71 V_rms_ per port) and loading with either sphere or body. |B|_10m_ patterns were shown in three different views: A side‐on view and views looking down either the service‐end or patient‐end.

Figure 8 shows the |B|_10m_ patterns in MRI systems at 0.25T and 0.5T (the two field strengths studied where the regulatory limits specify |B|_10m_ limits) for both sphere and body loads, in the absence of the Faraday shielding. At both fields, the |B|_10m_ pattern is symmetric for the spherical load but is asymmetric for the body load. For the body load, the peak‐|B|_10m_ of the 0.5T system is 2.8‐fold (9 dB) higher than that of the 0.25T system. The ratio of the peak‐|B|_10m_ for the body and sphere loads was 1.3 dB at 0.25T and increased to 1.9 dB at 0.5T. In the 0.25T and 0.5T systems with the body load, an RF pulse with a peak of 300 V_rms_ exceeds the regulatory limit by 7.1‐fold (36 dB) and 62.7‐fold (45 dB), respectively.

**FIGURE 9:**
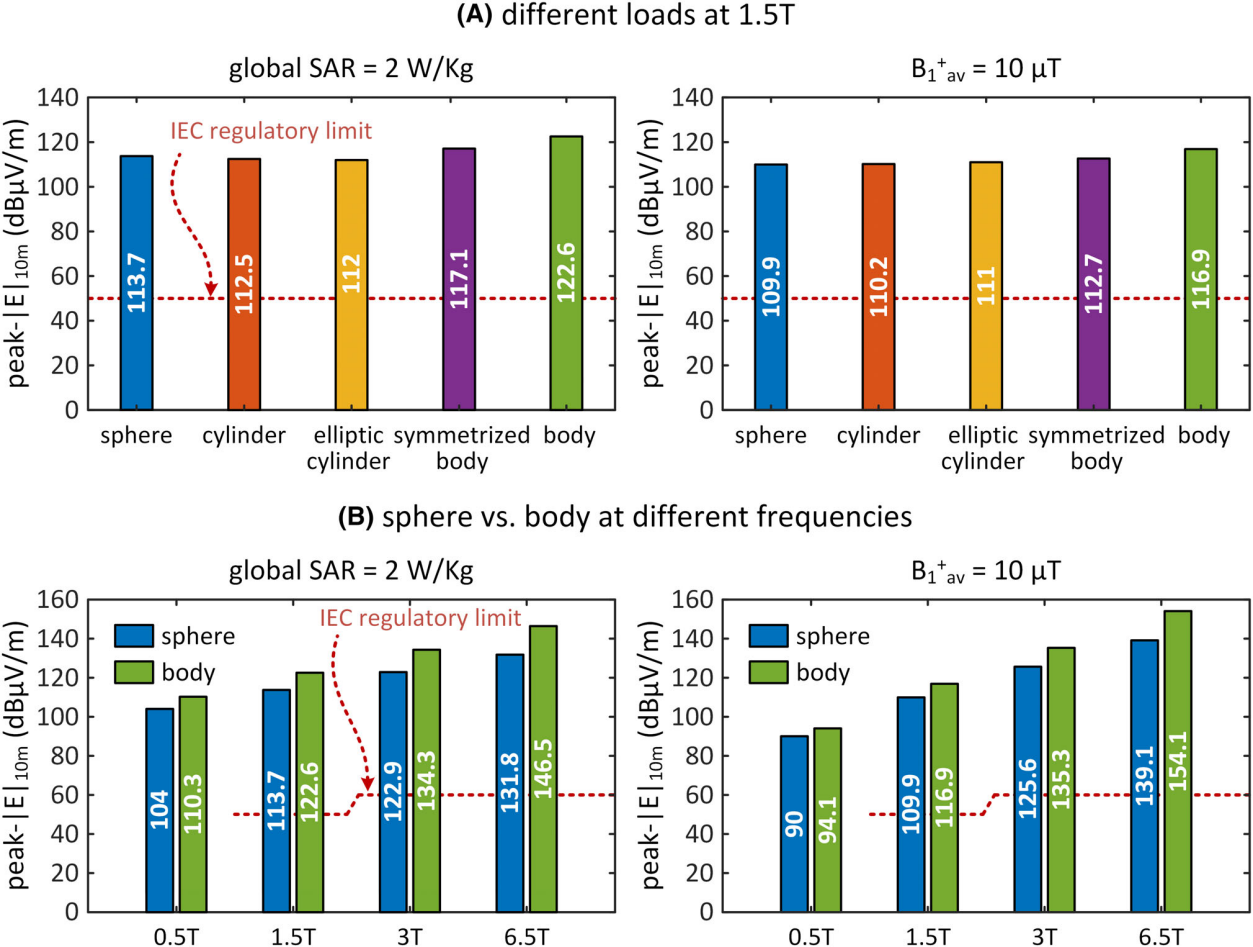
Peak‐|E|_10m_ expressed in dBμV/m for the CP body birdcage coil driven to achieve either 2 W/kg SAR in the irradiated section of the load with a 10% duty‐cycle CW RF input, or an average B_1_^+^ field of 10 μT in the central axial slice. The regulatory limit for peak‐|E|_10m_ is shown as a horizontal dashed line. (A) Peak‐|E|_10m_ for different loads at 1.5T. (B) Comparison of peak‐|E|_10m_ between sphere and body loads for 0.5T, 1.5T, 3T, and 6.5T systems.

Figure 9 plots the peak‐|E|_10m_ for all 5 loads at 1.5T, as well as for sphere and body loads at 0.5T, 1.5T, 3T, and 7T for power inputs corresponding to two realistic use scenarios. The coil inputs were adjusted to achieve either the maximum allowable global‐SAR (2 W/kg) at a 10% duty‐cycle or an average B_1_^+^ of 10 μT in the central axial slice. At 1.5 T under the constant global‐SAR condition, the peak‐|E|_10m_ value for the body load was at least 5.5 dB higher than for other loads. Under the constant B_1_^+^ condition, the peak‐|E|_10m_ of the body load was 4.2 dB higher. At all other field strengths, the body load also shows a higher peak‐|E|_10m_ than the sphere load. For the constant 2 W/kg global‐SAR, increasing B_0_ from 0.5T to 6.5T increases the peak‐|E|_10m_ by 27.8 dB for the sphere load and 36.2 dB for the body load. Under the constant B_1_^+^ condition, ramping from B_0_ from 0.5T to 6.5T increases the peak‐|E|_10m_ by 49.1 dB for the sphere load and 60 dB for the body load. Figure S5 shows the peak‐|E|_10m_ dependence on field strength is well‐fit by a second‐order polynomial.

**FIGURE 10:**
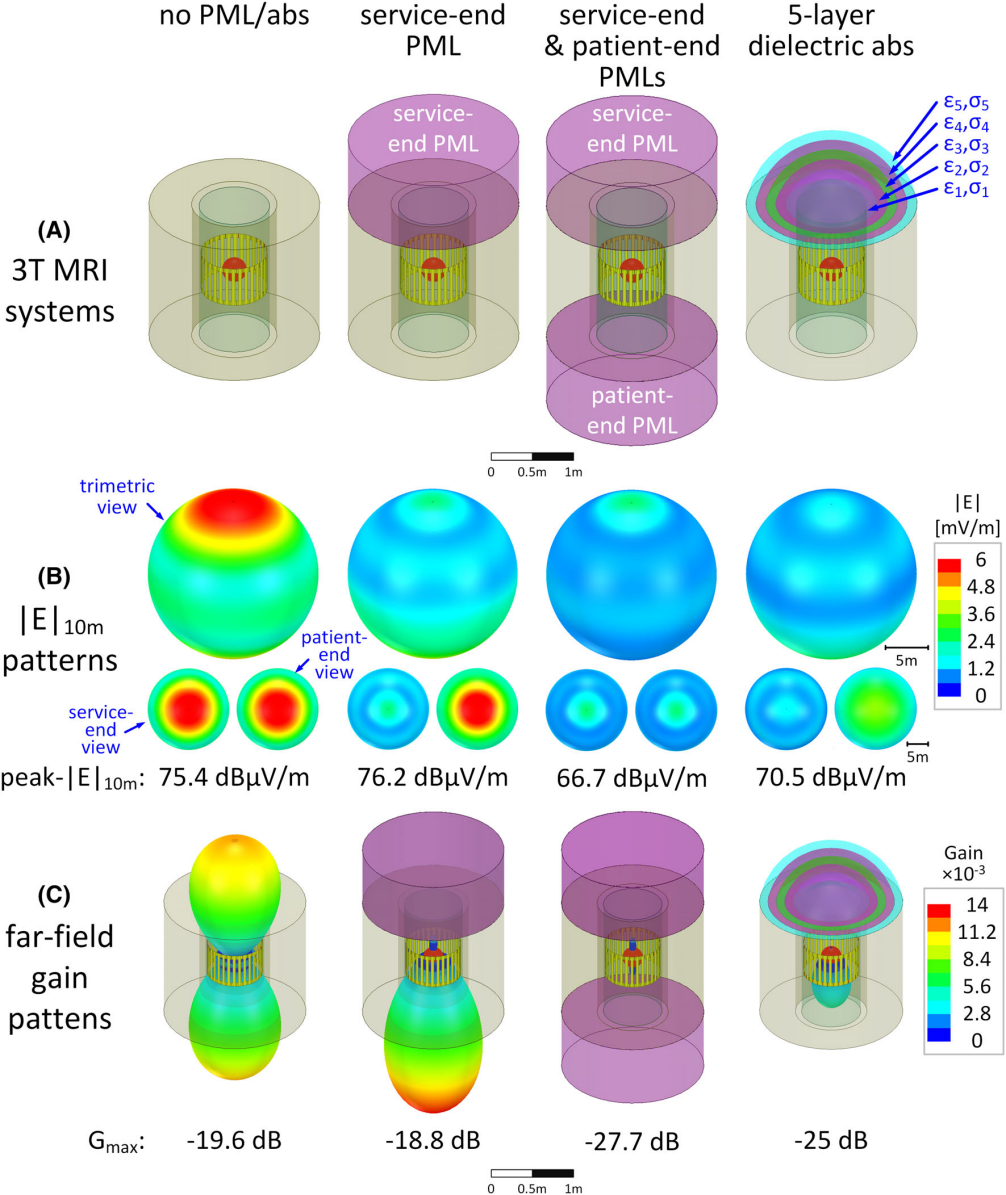
Analysis of four absorber configurations for a 3T MRI system without a Faraday shielded room, using a CP‐driven birdcage coil with 1 V_rms_ total input (0.71 V_rms_ per port) and a sphere load. (A) The configurations studied: No absorber, a Perfectly Matched Layer (PML) at the bore's service‐end, PMLs at both ends, and an optimized five‐layer domed dielectric absorber at the service‐end with relative permittivities (ε_r_) of 92.2, 111.6, 636.1, 1285.2, and 523.7 for layers 1 to 5 (inner to outer), each with a thickness of 101.6 mm and conductivities of 78, 186.9, 1100.7, 1482.4, and 507.2 S/m, respectively. (B) |E|_10m_ patterns, and (C) far‐field gain patterns.

Figure 10 shows the |E|_10m_ and far‐field gain patterns of a 3T MRI system with a spherical load, in the absence of the Faraday shielding, under different EM absorber configurations: no absorber, a disk‐shaped PML absorber positioned at the bore's service‐end, PMLs at both the service and patient‐ends, and an optimized five‐layer domed dielectric absorber at the service‐end. In the baseline configuration without any absorber, the |E|_10m_ and gain peaks are directed toward the bore's ends. Adding the service‐end PML reduced the |E|_10m_ and gain patterns at the service‐end by 8.3 dB and 7.5 dB, respectively, while increased them both by 0.8 dB at the patient‐end. This service‐end PML effectively absorbs normal waves but partially reflects non‐normal waves, altering surface currents on the bore, birdcage, RF‐shield, and body in a destructive manner, thereby slightly increasing the |E|_10m_ in the patient‐end direction. With PMLs at both ends, the peak‐|E|_10m_ and *G*_*max*_ reduced by 8.7 dB and 8.1 dB, respectively. Fully covering the scanner with PMLs could absorb radiation significantly but is impractical for patient imaging. Optimization of the five‐layer domed absorber resulted in relative permittivities of 92.2, 111.6, 636.1, 1285.2, and 523.7 and conductivities of 78, 186.9, 1100.7, 1482.4, and 507.2 S/m for layers one (innermost‐layer) to five (outermost‐layer). Compared to the baseline configuration, this absorber reduced the service‐end and patient‐end peaks of the |E|_10m_ pattern by 13.5 dB and 4.9 dB, respectively, and the service‐end and patient‐end peaks of the gain pattern by 12.2 dB and 5.4 dB, respectively. The dielectric EM absorber outperforms the service‐end PML configuration by not only absorbing outgoing EM‐fields but also reflecting some back into the bore, causing destructive interference that cancels EM‐fields in the patient‐end direction.

## 4 DISCUSSIONS AND CONCLUSIONS

The high siting costs associated with traditional MRI installations pose a considerable challenge to the widespread accessibility of MRI technology. This issue is receiving increasing scrutiny in the field, as evidenced by the increased interest in low‐field MR technology for use in both developed countries and worldwide.^2^, ^3^, ^10^, ^46^, ^47^, ^48^, ^49^, ^50^ In particular, the simpler siting process of low‐field portable systems, including operation without a Faraday shielding, is a major advantage but extending this to traditional superconducting clinical systems is challenging. While low‐field MRI systems improve access and affordability (including portability) at the expense of sensitivity, superconducting magnet‐based systems with *B*_*0*_ ≥ 0.5T will likely remain the primary tool of diagnostic radiology services. Because of this, this study's focus centers on characterizing the RF emission of traditional superconducting MRI systems as a necessary first step toward the development of alternative, cheaper EM radiation mitigation strategies.

Our simulations characterize these EM radiation patterns and levels for a representative superconducting magnet‐based MR scanner geometry operated without a Faraday shielding as a starting point for contemplating cost‐effective mitigation strategies. This simulation‐based approach allows us to efficiently assess a broad range of scenarios and parameters, such as load symmetry and field strength, which would be challenging and costly to investigate experimentally. The simulations could provide a robust foundation for understanding RF emissions and guiding the design of mitigation strategies. While this study focuses on simulation‐based characterization, future work must include experimental studies to validate these strategies further and confirm their applicability in clinical settings. Due to the difficulty and expense of dedicating an operational scanner to a site without Faraday shielding, this process will likely start with a mock‐scanner with an operational body coil.

At mid‐field (1T–1.5T), the donut‐shaped radiation pattern seen for the spherical load follows the shape expected for a current loop induced around the ends and waste of the magnet and/or shield. As the frequency is increased, traveling wave effects in the bore appear to take over, producing a gain pattern peaked at the bore‐ends. This occurs at a lower frequency than might be expected from the cutoff frequency of a cylindrical waveguide with the bore dimensions (this cutoff frequency is 195 MHz for the bore geometry modelled). The high‐field radiation out the bore field is also strongly affected by the body symmetry. With the body load present, the power emerging from the foot‐end of the magnet dominates, suggesting the body significantly facilitates the wave‐guide effect (Figure 7). At low‐field (0.25T and 0.5T), the pattern returns to a subdued version of this bore‐end dominant pattern as opposed to simply a weakening version of the 1T donut pattern. The isotropic pattern at 0.75T and the combined donut and bore‐end pattern at 1.75T can be interpreted as two types of transition patterns.

As expected, the |E|_10m_ and |B|_10m_ generated by the body RF coil for realistic transmit levels exceed regulatory limits for all field strengths studied (0.25T to 6.5T) and require significant mitigation. The total radiated power and peak‐|E|_10m_ increase dramatically with the field strength. Although 10 m fields are greater at higher field strength (3T and above), we also show that their patterns are potentially easier to cancel because power primarily radiates out the bore‐ends rather than from the waist of the scanner (Figures 6 and 7), suggesting that mitigation efforts focused near these regions could effectively disrupt wave propagation. These findings underscore the importance of understanding the driving physics of RF emissions to develop effective mitigation strategies. By leveraging these insights, future work can focus on optimizing system geometry, absorber or cancellation source placement, and other mitigation techniques to improve compliance with EMC standards.

Our simulations demonstrate that radiated power and absorbed power by the spherical load both increase with field strength for constant input power (Figure 5). Previous studies have indicated that RF power requirements to achieve a specific flip‐angle within the load vary with field strength,^51^, ^52^, ^53^, ^54^, ^55^ influenced by factors like coil type and load properties. At higher B_0_, EM interactions between the coil and load dominate, complicating the relationship. For example, quasistatic models predict a square‐law relationship between the required power and B_0_, while full‐wave models introduce additional resonances and dependencies on coil and load interactions.^55^ Despite these variations, a general trend is higher frequencies require more RF power to achieve the same flip‐angle. Therefore, achieving the same flip‐angle is expected to produce more radiation than depicted in Figure 5. This complexity underscores the need for detailed simulation studies like ours to assess radiation characteristics comprehensively. Figure 9B illustrates this trend for both spherical and body loads under a 90° excitation using a 0.6 ms square pulse.

EM radiation patterns and levels depend on factors such as body size, shape, posture, and tissue dielectric properties, as well as the frequency and polarization of the incident field. This study focuses on the most common clinical scanner geometry: a solenoidal superconducting magnet, a CP‐mode birdcage body coil, and a cylindrical RF‐shield. While the exact levels and patterns will likely be modulated by differences in the exact geometry, we acknowledge that any manufacturer wishing to operate their scanner without a Faraday room will need to simulate (and experimentally validate) their exact geometry. Nonetheless, the findings provide critical insights into shielding‐free MRI systems.

The radiated EM‐field patterns presented in this study are evaluated at a 10 m distance from the scanner. Radiated power distribution is influenced by the transition between the near‐field and far‐field regions, determined by the system's largest dimension and the operating wavelength. At closer distances, the fields are predominantly shaped by reactive and radiative coupling within the near‐field zone. In the far‐field, the radiated power decreases proportionally to the square of the inverse of the distance due to spherical wavefront propagation. The IEC standards specify radiated field limits based on peak EM‐field levels at nominal distances of 3, 10, or 30 m. The regulatory limits given for these distances follow a *1/r* ^*2*^ scaling, and the regulators allow the user to choose the distance. For this study, we selected a measurement distance of 10 m to balance practical considerations. We sought the smallest simulation region size that would ensure the results were evaluated in a far‐field regime (a distance greater than *2D* ^*2*^ */λ*) for the *D* = 2.5 m scanner structure. Figure S6 shows the |E|‐field levels and patterns on 3, 5, 10, 15, 20, and 30 m radius spheres.

While we sought to illuminate the potential Tx radiation at 7T, the maximum field strength we could accurately model (tune the birdcage coil) was 6.5T. At lower field strengths (B_0_ < 4T), the tuning capacitors decrease approximately inversely with the square of the field strength (*c*_*t*_ *∝ 1/B*_*0*_^*2*^), requiring smaller and smaller tuning capacitors for higher B_0_. However, when the capacitance becomes on the order of the stray capacitance of the coil's conductive structures, further reducing the lumped‐element capacitor no longer has the desired effect on the coil tuning. Therefore, 6.5T was the highest field for which we could utilize the chosen birdcage and RF‐shield geometry (which was originally intended for a 3T scanner).

Beyond the dependency on field strength, we found that load asymmetry significantly influences radiation patterns, and the realistic body models exhibited the most anisotropic patterns. Figure S7 shows that the male and female body models have similar radiation patterns and levels. While further analysis over a population is needed, this suggests that mitigation strategies are likely resilient to variations in body shapes and may not require a patient‐specific approach. However, only one body position (head at isocenter) was studied. Figure S8 examines the impact of adding realistic tissue heterogeneity to the body model by comparing uniform and anatomically detailed models. While heterogeneity introduces a 0.6 dB increase in peak‐|E|_10m_ for the detailed model, the overall radiation levels and patterns remain consistent. These findings support using uniform models as an efficient and reliable starting point for simulations, with detailed models reserved for refining results in future investigations.

Precise numerical modeling was needed, particularly at low and mid‐field strengths since the radiated power is lower than the power absorbed by conductive structures or the load itself. For example, at 0.25T, the radiated‐to‐accepted power ratio was only 7.9 × 10^−5^, although it still exceeded regulatory levels for realistic excitations. ANSYS HFSS, a widely utilized tool for accurate simulations, achieves this by implementing refined meshes across structural components. An adaptive meshing process adjusts the mesh throughout the simulation domain based on field variations, ensuring accurate capture of EM behavior. This refinement is not limited to port regions but extends to areas with rapid field changes or intricate geometrical features. Accurately modeling radiation requires fine meshes on both the surface and volume of the radiating region. However, uniformly fine meshes increase the overall mesh count substantially, making it computationally impractical. To balance accuracy and computational efficiency, we conducted supplementary simulations (Figures S9‐S14) to optimize mesh sizes for different MRI components. All simulations converged within two to four adaptive iterations, with total mesh counts ranging from 5.2 million to 11 million.

A five‐layer EM absorber was tested at 3T as a preliminary EM mitigation solution. This reduced the service‐end peak‐|E|_10m_ by 13.5 dB and patient‐end peak‐|E|_10m_ by 4.9 dB, showing promise for practical implementation. However, even with an input voltage of 1 V_rms_, the patient‐end peak‐|E|_10m_ exceeds the IEC regulation by 10.5 dB and further mitigation is therefore needed. The optimization of this absorber was limited by the assumption of spherical curvature layers with a fixed uniform thickness, and only the conductivity and relative permittivity of the layers were adjusted to reduce peak‐|E|_10m_. To enhance their mitigation efficiency, future work should consider more degrees of freedom, including non‐uniform geometrical features, to refine the optimization process. Additionally, a wider range of RF absorber designs, beyond the simple layered absorbers tested here should be considered and may provide better attenuation performance, particularly at higher field strengths. Investigating the placement of absorbers both inside and around the bore, in addition to the bore service‐end, could further improve mitigation.

This study establishes the primary radiation characteristics and levels of conventional birdcage coil excitation in MRI systems operating without Faraday shielding but multiple lines of approach exist for mitigating Tx radiation levels. Progress has recently been reported in using an array of auxiliary transmitters outside a clinical whole‐body 0.55T scanner to actively cancel |H|_10m_‐fields.^56^ While promising, achieving EM compliance requires a sufficiently large number of auxiliary transmitters (32 or more), and practical challenges remain in precisely controlling their phases and amplitudes to adapt to patient‐specific variations. Additionally, alternative RF coil designs, such as parallel transmission (pTx) arrays, offer a potential pathway for shaping spin excitation with controlled |E|_10m_‐fields.^57^, ^58^ Transmit arrays (pTx) have been extensively used to shape spin excitation while explicitly controlling E‐fields inside the body (local‐SAR).^31^, ^59^ The same framework can be extended beyond local‐SAR control to mitigate |E|_10m_‐fields.^58^ Additionally, MRI sequence and protocol optimizations—such as RF pulse shaping and amplitude modulation—could reduce input peak RF levels and minimize radiated RF leakage while maintaining imaging performance. All these techniques, when combined with more sophisticated optimized EM absorbers, could provide a comprehensive strategy toward achieving practical unshielded MRI operation. Our work establishes the primary radiation characteristics of conventional birdcage excitation, future studies will focus on exploring and evaluating mitigation methods.

## Supporting information


**Table S1.** ANSYS HFSS's human body average electrical properties as a function of field strength.
**Figure S1.** |E|_10m_ patterns for a 1.5 T MRI system under three conditions: (1) without a Faraday shielded room, (2) with a Faraday shielded room, and (3) with a Faraday shielded room when the door is open. Each system comprises a magnet, an RF‐shield, an RF body CP‐driven birdcage coil with 1V_rms_ total input (0.71V_rms_ per port) loaded with a uniform body model. The E‐field magnitude at a 10 m radius is painted on the sphere in dBμV/m units. **(A)** Details and dimensions, and (B) |E|_10m_ patterns. The Faraday shielded room reduced the E‐field at 10 m by 84 dB under closed‐door condition.
**Figure S2.** The data from Figure 2 presented from the service‐end view. |E|_10m_ and gain patterns (service‐end view) for a 1.5 T MRI system without a Faraday shielded room under various loads in the CP excitation mode driven with 1 V_rms_ total input (0.71 V_rms_ per port). (A) |E|_10m_ patterns and (B) far‐field gain patterns.
**Figure S3.** |B_1_^+^| maps for the 1.5 T body coil operated in the CP mode driven with 1 V_rms_ total input (0.71 V_rms_ per port) for each of the 5 loads studied: sphere, cylinder, elliptical cylinder, symmetrized body, and body, shown in the central (A) axial view and (B) sagittal view.
**Figure S4.** |B_1_^+^| maps within the central axial and sagittal planes of an unshielded MRI system with a birdcage coil driven in the CP excitation mode with 1 V_rms_ total input (0.71 V_rms_ per port) loaded with a sphere as a function of field strength.
**Figure S5.** Second‐order polynomial fit to the peak‐|E|_10m_ as a function of magnetic field strength (B_0_) for an unshielded MRI system using the CP‐driven birdcage coil with either sphere or body loads. The birdcage coil is driven to achieve either (A) 1 V_rms_ total input or (B) an average B_1_^+^ of 10 μT in the central axial slice.
**Figure S6.** |E|‐field patterns (trimetric view) for a 1.5 T MRI scanner without a Faraday shielded room, using a uniform male body load and CP excitation mode driven with 1 V_rms_ total input (0.71 V_rms_ per port). E‐field magnitudes are shown at distances of 3 m, 5 m, 10 m, 15 m, 20 m, and 30 m.
**Figure S7.** |E|_10m_ and gain patterns (trimetric view) for an unshielded 1.5 T MRI system with the male and female body loads and CP excitation mode driven with 1 V_rms_ total input (0.71 V_rms_ per port). (A) body models, (B) |E|_10m_ patterns, and (C) far‐field gain patterns.
**Figure S8.** |E|_10m_ and gain patterns (trimetric view) for an unshielded 1.5 T MRI system with the uniform and detailed male body loads and CP excitation mode driven with 1 V_rms_ total input (0.71 V_rms_ per port). (A) uniform vs. detailed body models, (B) |E|_10m_ patterns, and (C) far‐field gain patterns.
**Figure S9.** Normalized power analysis for an unshielded 1.5 T MRI system with a CP‐driven birdcage coil loaded with the body model for different maximum mesh sizes on the radiation box surface, i.e., < 125 mm, < 250 mm, and <375 mm. The bars show the radiated power, and the power dissipated in the birdcage coil, RF shield, and body load. These values are expressed as percentages of the forward power (power accepted by the birdcage coil).
**FIGURE S10.** Normalized power analysis of an unshielded 1.5 T MRI system with a CP‐driven birdcage coil and loaded with the body model for varying maximum mesh sizes inside the radiation box surface: 250 mm, 500 mm, and without limitation. The bars represent the radiated power, and the power dissipated in the birdcage coil, RF shield, and body load. These values are expressed as percentages of the forward power (power accepted by the birdcage coil).
**Figure S11.** Normalized power analysis for an unshielded 1.5 T MRI system using a CP‐driven and body‐loaded birdcage coil with varying maximum birdcage coil mesh sizes (2 mm, 4 mm, and 6 mm). Bars indicate the radiated power and the power dissipation in the birdcage coil, RF shield, and body load, expressed as percentages of the forward power accepted by the birdcage coil.
**Figure S12.** Normalized power analysis of an unshielded 1.5 T MRI system using a CP‐driven, body‐loaded birdcage coil with different maximum mesh sizes on the RF shield: 10 mm, 20 mm, and 30 mm. The bars represent the radiated power and the power dissipation within the birdcage coil, RF shield, and body load as percentages of the forward power accepted by the birdcage coil.
**Figure S13.** Normalized power analysis of an unshielded 1.5 T MRI system using a CP‐driven birdcage coil loaded with the body for 3 different maximum mesh sizes on the magnet surface (20, 40, and 60 mm). The bars represent the radiated power, and the power dissipated in the birdcage coil, RF shield, and body load as percentages of the forward power (power accepted by the birdcage coil).
**Figure S14.** Normalized power analysis for an unshielded 1.5 T MRI system with a CP‐driven birdcage coil loaded with the body model using different maximum mesh sizes within the body model (<5, <10, and <15 mm). The bars show the radiated power and the power dissipation in the birdcage coil, RF shield, and body load expressed as percentages of the forward power (power accepted by the birdcage coil).
