# Supplementary material for: Simulated radiation levels and patterns of MRI without a Faraday shielded room
Source: Magn Reson Med. 2025 Mar 17;94(2):835–51. doi: 10.1002/mrm.30499 (PMC12137786; doi:10.1002/mrm.30499)
Supplement: Supplementary file 1 — Table S1. ANSYS HFSS's human body average electrical properties as a function of field strength. Figure S1. |E|10m patterns for a 1.5 T MRI system under three conditions: (1) without a Faraday shielded room, (2) with a Faraday shielded room, and (3) with a Faraday shielded room when the door is open. Each system comprises a magnet, an RF‐shield, an RF body CP‐driven birdcage coil with 1Vrms total input (0.71Vrms per port) loaded with a uniform body model. The E‐field magnitude at a 10 m radius is painted on the sphere in dBμV/m units. (A) Details and dimensions, and (B) |E|10m patterns. The Faraday shielded room reduced the E‐field at 10 m by 84 dB under closed‐door condition. Figure S2. The data from Figure 2 presented from the service‐end view. |E|10m and gain patterns (service‐end view) for a 1.5 T MRI system without a Faraday shielded room under various loads in the CP excitation mode driven with 1 Vrms total input (0.71 Vrms per port). (A) |E|10m patterns and (B) far‐field gain patterns. Figure S3. |B1 +| maps for the 1.5 T body coil operated in the CP mode driven with 1 Vrms total input (0.71 Vrms per port) for each of the 5 loads studied: sphere, cylinder, elliptical cylinder, symmetrized body, and body, shown in the central (A) axial view and (B) sagittal view. Figure S4. |B1 +| maps within the central axial and sagittal planes of an unshielded MRI system with a birdcage coil driven in the CP excitation mode with 1 Vrms total input (0.71 Vrms per port) loaded with a sphere as a function of field strength. Figure S5. Second‐order polynomial fit to the peak‐|E|10m as a function of magnetic field strength (B0) for an unshielded MRI system using the CP‐driven birdcage coil with either sphere or body loads. The birdcage coil is driven to achieve either (A) 1 Vrms total input or (B) an average B1 + of 10 μT in the central axial slice. Figure S6. |E|‐field patterns (trimetric view) for a 1.5 T MRI scanner without a Faraday shielded room, using a uniform male body l [file MRM-94-835-s001.docx]

**Simulated radiation levels and patterns of MRI without a Faraday shielded room**

*Authors:* Ehsan Kazemivalipour^1,2^, Bastien Guerin^1,2^, and Lawrence L. Wald^1,2,3^

^1^A. A. Martinos Center for Biomedical Imaging, Department of Radiology, Massachusetts General Hospital, Charlestown, Massachusetts, USA

^2^Harvard Medical School, Boston, Massachusetts, USA
^3^Harvard-MIT Division of Health Sciences Technology, Cambridge, Massachusetts, USA

Here are supplementary results not included in the main text of the manuscript.

**SUPPORTING INFORMATION TABLE S1:**

| **Table S1** – ANSYS HFSS’s human body average electrical properties as a function of field strength | | |
| --- | --- | --- |
| **field strength** | **conductivity (σ)** | **relative permittivity (ε_r_)** |
| 0.25T | 0.3 S/m | 99.67 |
| 0.5T | 0.31 S/m | 92.67 |
| 0.75T | 0.32 S/m | 85.33 |
| 1T | 0.34 S/m | 78.33 |
| 1.25T | 0.35 S/m | 71.33 |
| 1.5T | 0.36 S/m | 64 |
| 1.75T | 0.37 S/m | 57 |
| 2T | 0.38 S/m | 50 |
| 2.5T | 0.4 S/m | 39.94 |
| 3T | 0.41 S/m | 39.72 |
| 4T | 0.43 S/m | 39.3 |
| 6.5T | 0.48 S/m | 38.23 |

**SUPPORTING INFORMATION FIGURE S1:**


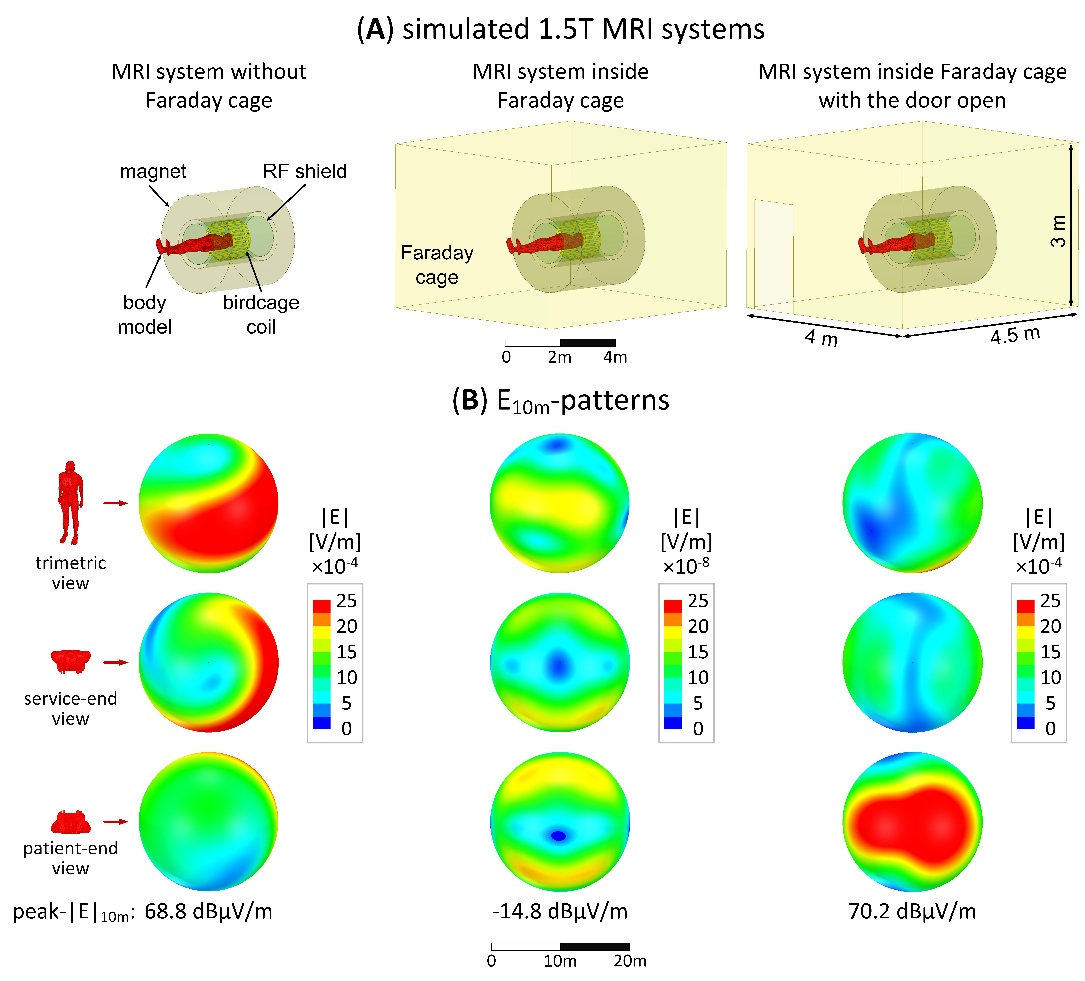


**Figure S1** – |E|_10m_ patterns for a 1.5T MRI system under three conditions: (1) without a Faraday shielded room, (2) with a Faraday shielded room, and (3) with a Faraday shielded room when the door is open. Each system comprises a magnet, an RF-shield, an RF body CP-driven birdcage coil with 1V_rms_ total input (0.71V_rms_ per port) loaded with a uniform body model. The E-field magnitude at a 10 m radius is painted on the sphere in dBµV/m units. **(A)**Details and dimensions, and (**B**) |E|_10m_ patterns. The Faraday shielded room reduced the E-field at 10 m by 84 dB under closed-door condition.

**SUPPORTING INFORMATION FIGURE S2:**


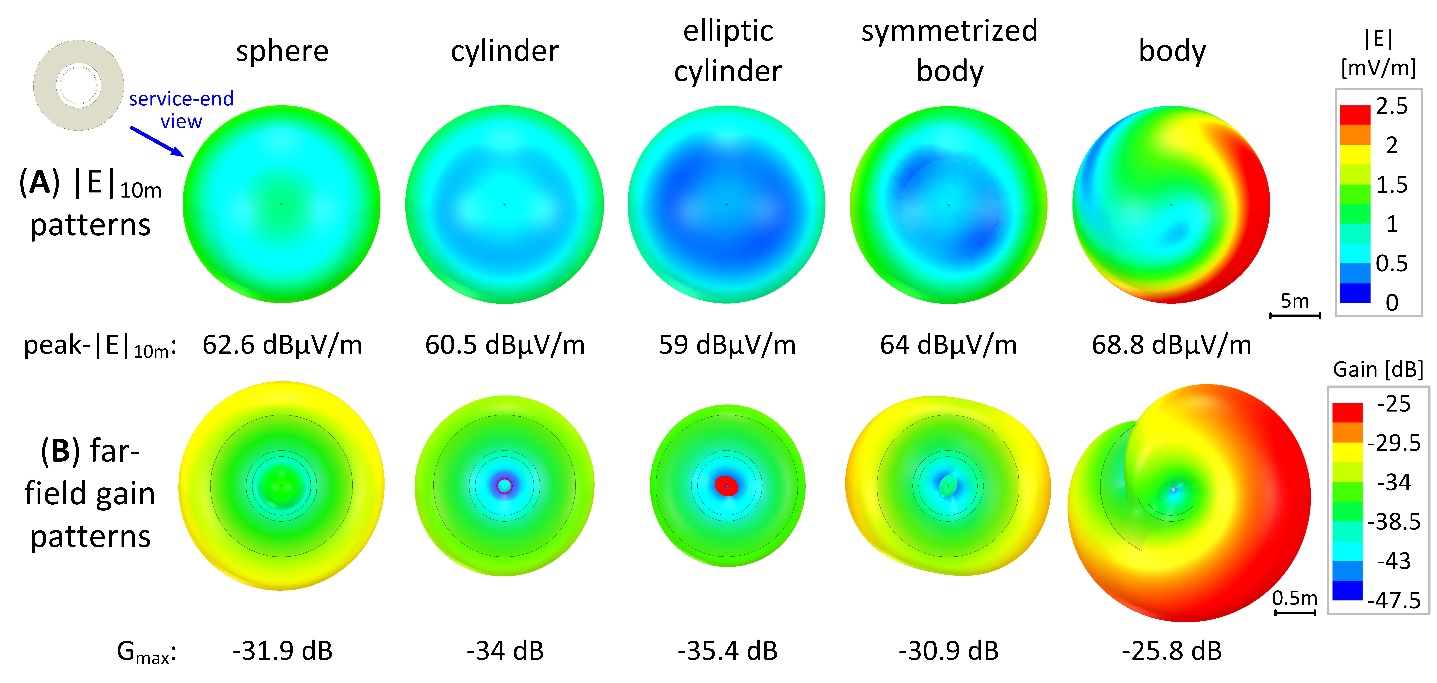


**Figure S2** – The data from Figure 2 presented from the service-end view. |E|_10m_ and gain patterns (service-end view) for a 1.5T MRI system without a Faraday shielded room under various loads in the CP excitation mode driven with 1 V_rms_ total input (0.71 V_rms_ per port). (**A**) |E|_10m_ patterns and (**B**) far-field gain patterns.

**SUPPORTING INFORMATION FIGURE S3:**


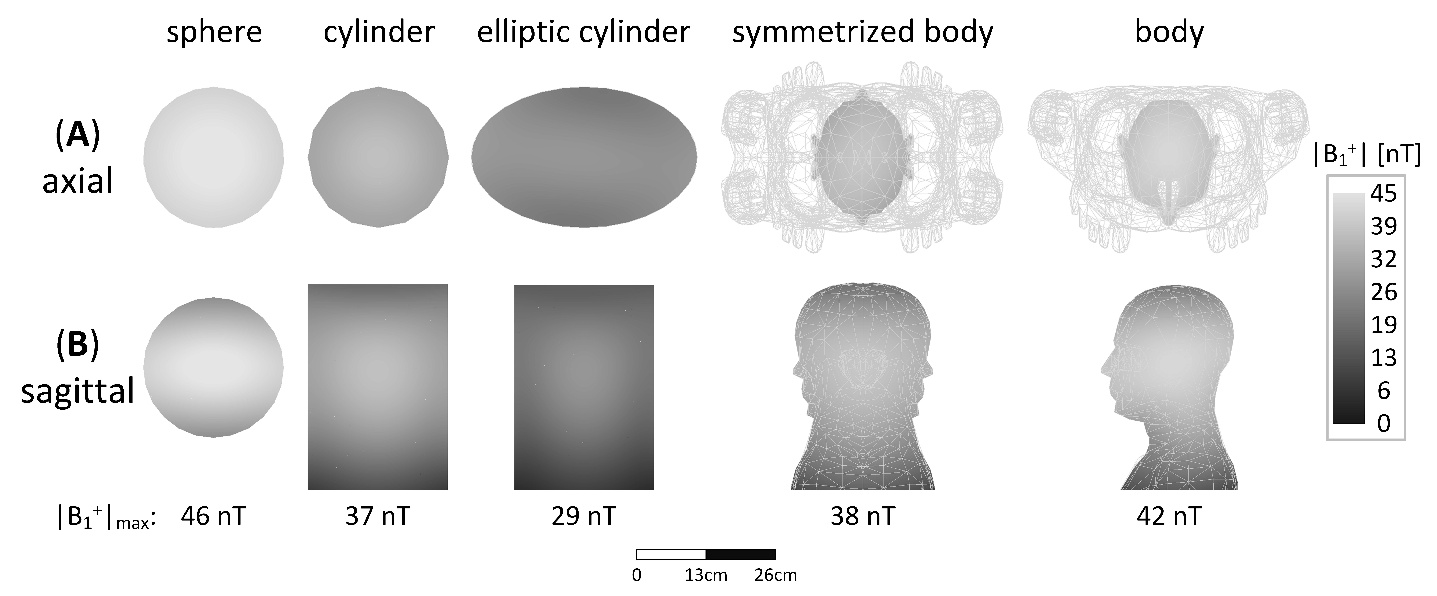


**Figure S3** – |B_1_^+^| maps for the 1.5T body coil operated in the CP mode driven with 1 V_rms_ total input (0.71 V_rms_ per port) for each of the 5 loads studied: sphere, cylinder, elliptical cylinder, symmetrized body, and body, shown in the central (**A**) axial view and (**B**) sagittal view.

**SUPPORTING INFORMATION FIGURE S4:**


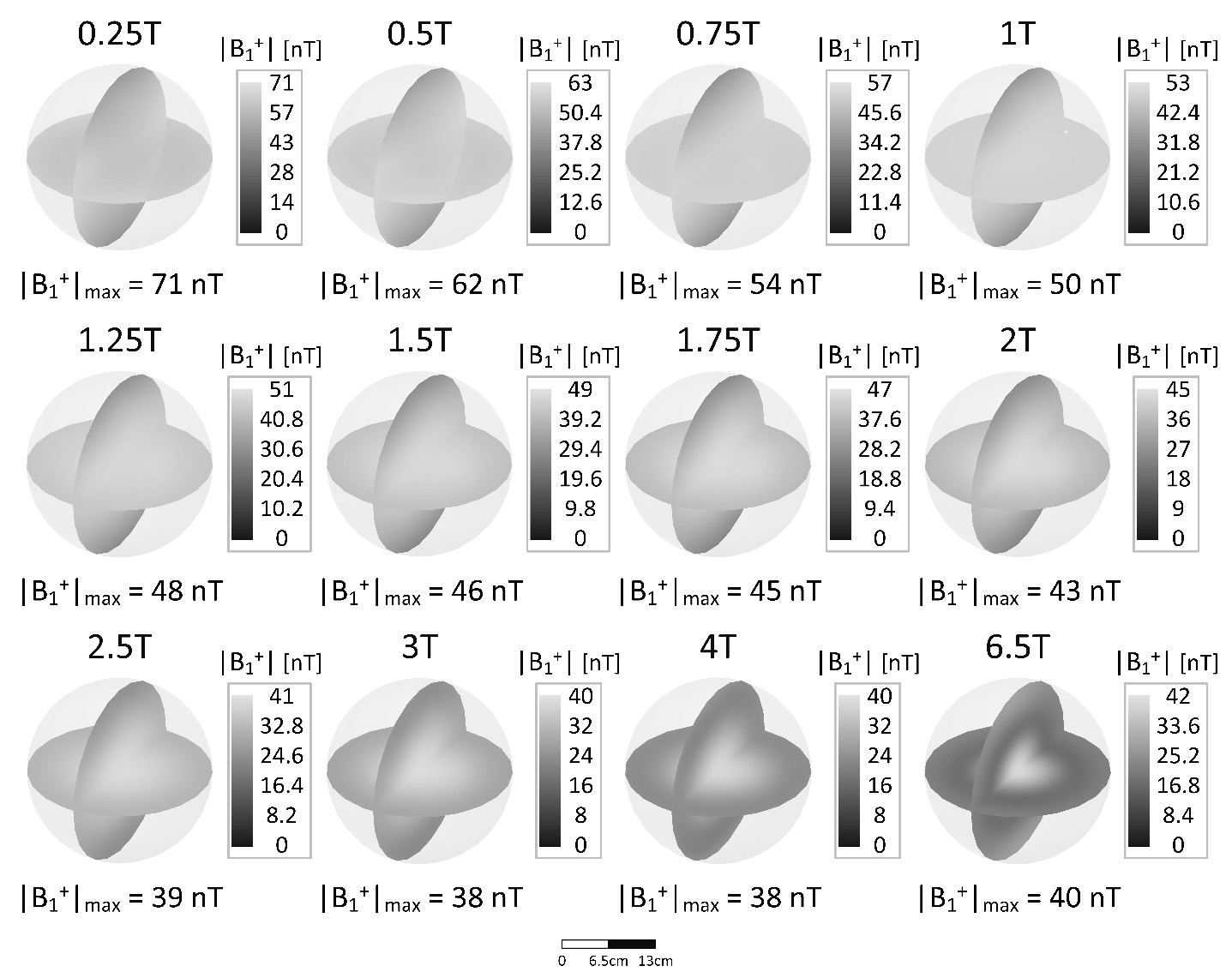


**Figure S4** – |B_1_^+^| maps within the central axial and sagittal planes of an unshielded MRI system with a birdcage coil driven in the CP excitation mode with 1 V_rms_ total input (0.71 V_rms_ per port) loaded with a sphere as a function of field strength.

**SUPPORTING INFORMATION FIGURE S5:**


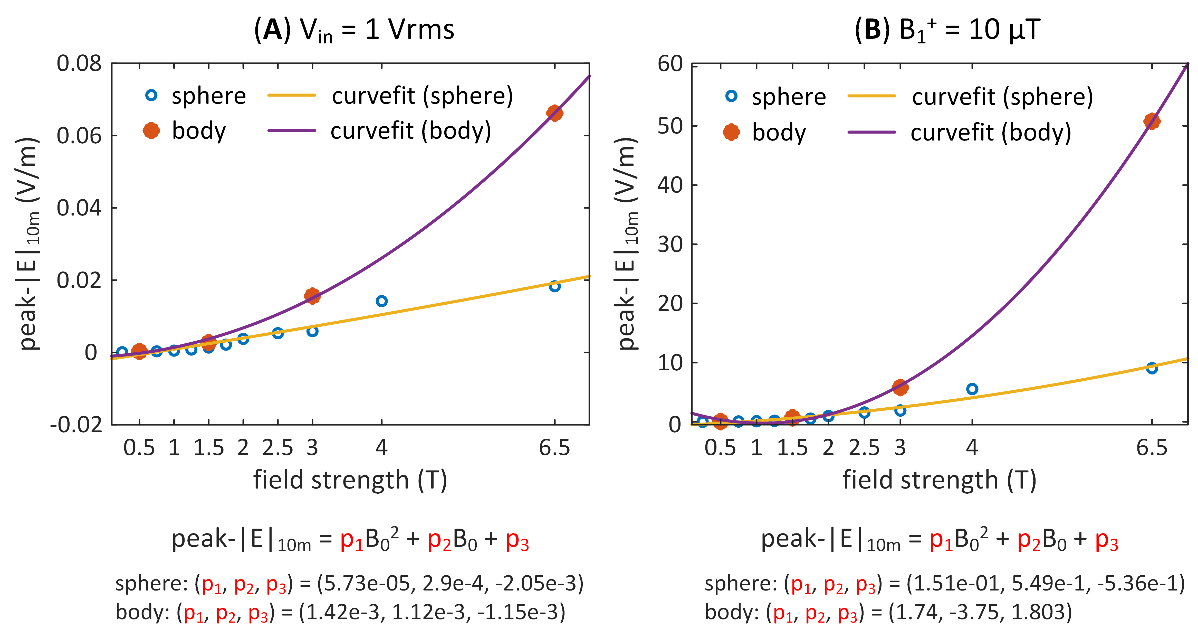


**Figure S5** – Second-order polynomial fit to the peak-|E|_10m_ as a function of magnetic field strength (B_0_) for an unshielded MRI system using the CP-driven birdcage coil with either sphere or body loads. The birdcage coil is driven to achieve either (**A**) 1 Vrms total input or (**B**) an average B_1_^+^ of 10 µT in the central axial slice.

**SUPPORTING INFORMATION FIGURE S6:**


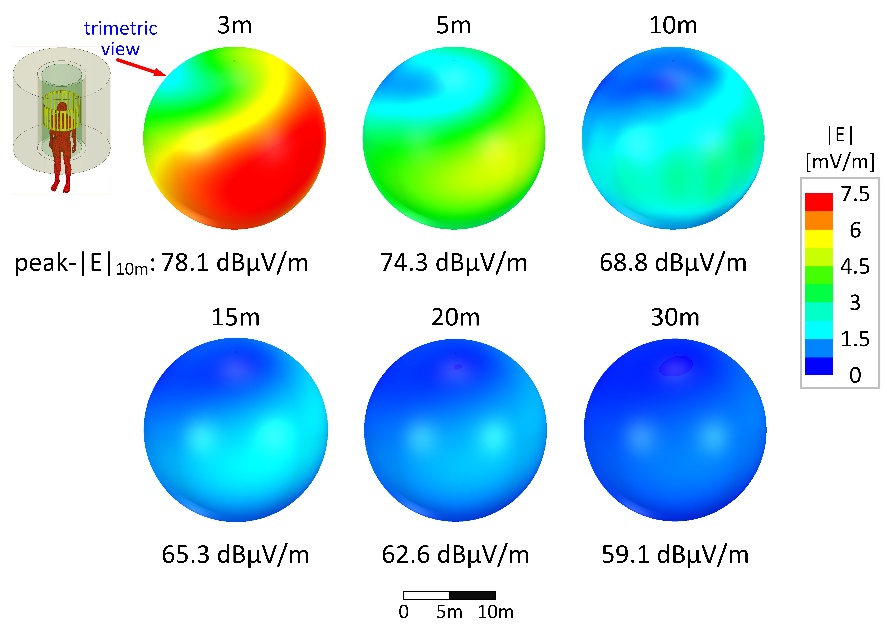


**Figure S6** – |E|-field patterns (trimetric view) for a 1.5T MRI scanner without a Faraday shielded room, using a uniform male body load and CP excitation mode driven with 1 V_rms_ total input (0.71 V_rms_ per port). E-field magnitudes are shown at distances of 3 m, 5 m, 10 m, 15 m, 20 m, and 30 m.

**SUPPORTING INFORMATION FIGURE S7:**


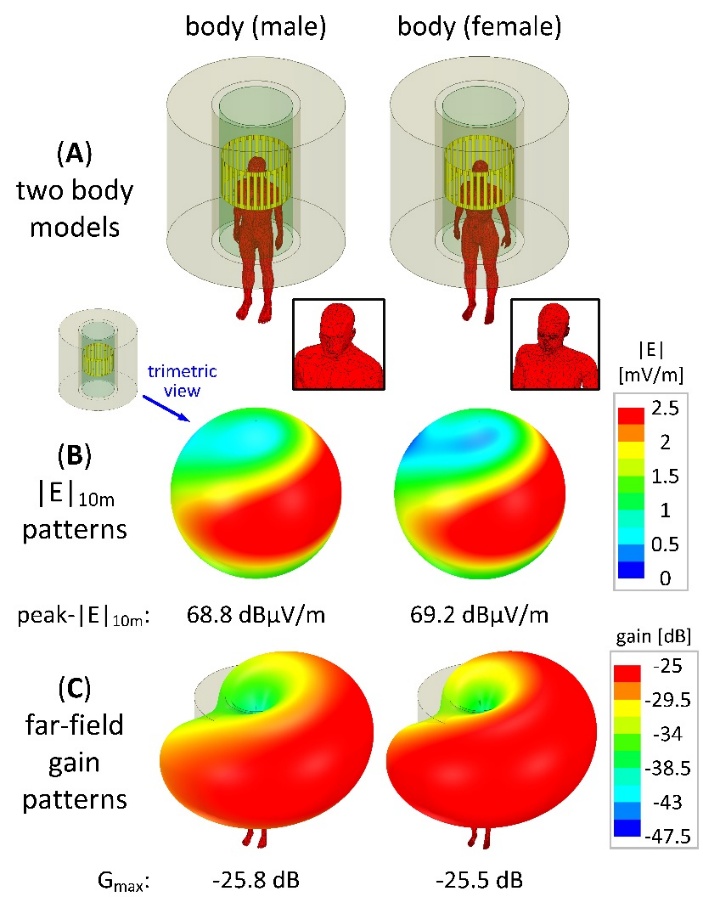


**Figure S7** – |E|_10m_ and gain patterns (trimetric view) for an unshielded 1.5T MRI system with the male and female body loads and CP excitation mode driven with 1 V_rms_ total input (0.71 V_rms_ per port). (**A**) body models, (**B**) |E|_10m_ patterns, and (**C**) far-field gain patterns.

**SUPPORTING INFORMATION FIGURE S8:**


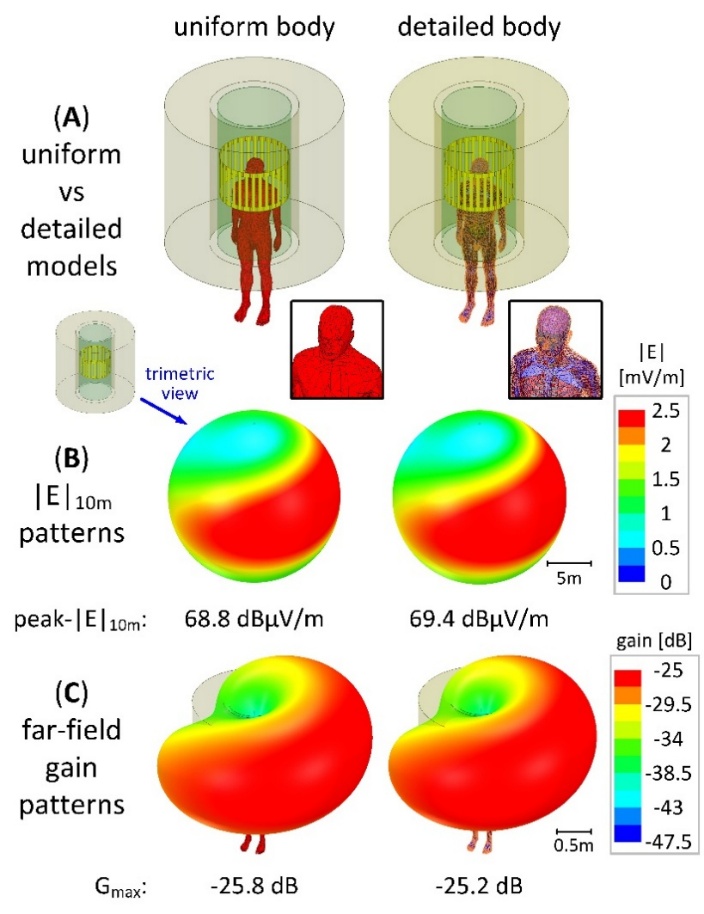


**Figure S8** – |E|_10m_ and gain patterns (trimetric view) for an unshielded 1.5T MRI system with the uniform and detailed male body loads and CP excitation mode driven with 1 V_rms_ total input (0.71 V_rms_ per port). (**A**) uniform vs detailed body models, (**B**) |E|_10m_ patterns, and (**C**) far-field gain patterns.

**SUPPORTING INFORMATION FIGURE S9:**


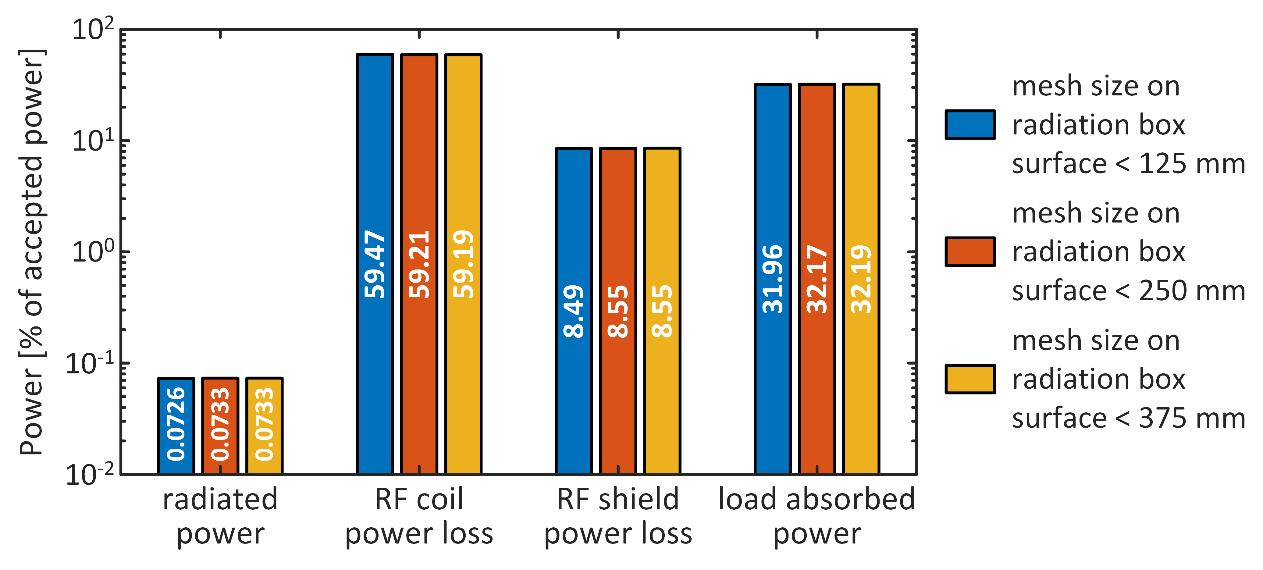


**Figure S9** – Normalized power analysis for an unshielded 1.5T MRI system with a CP-driven birdcage coil loaded with the body model for different maximum mesh sizes on the radiation box surface, i.e., < 125 mm, < 250 mm, and < 375 mm. The bars show the radiated power and the power dissipated in the birdcage coil, RF shield, and body load. These values are expressed as percentages of the forward power (power accepted by the birdcage coil).

**SUPPORTING INFORMATION FIGURE S10:**


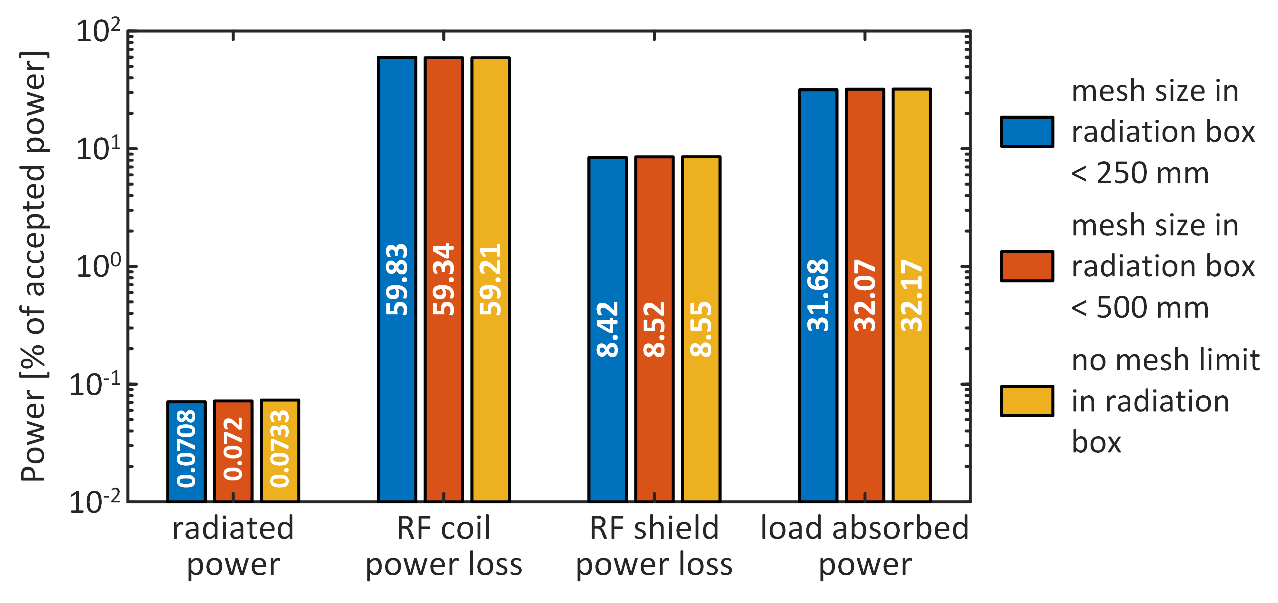


**Figure S10** – Normalized power analysis of an unshielded 1.5T MRI system with a CP-driven birdcage coil and loaded with the body model for varying maximum mesh sizes inside the radiation box surface: 250 mm, 500 mm, and without limitation. The bars represent the radiated power and the power dissipated in the birdcage coil, RF shield, and body load. These values are expressed as percentages of the forward power (power accepted by the birdcage coil).

**SUPPORTING INFORMATION FIGURE S11:**


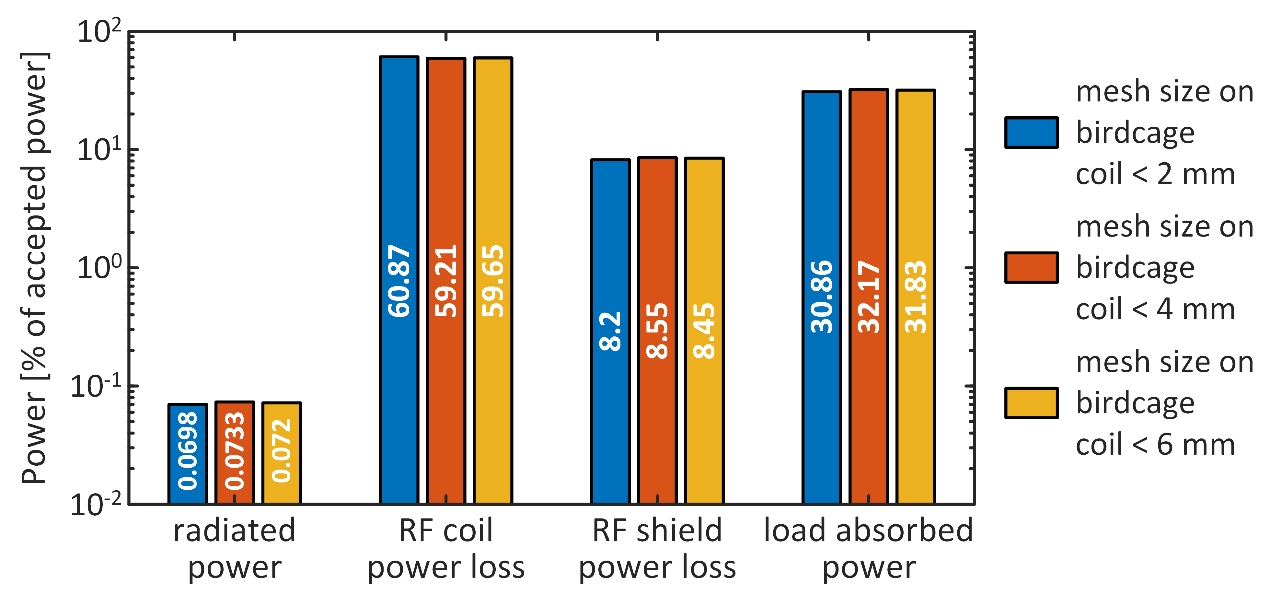


**Figure S11** – Normalized power analysis for an unshielded 1.5T MRI system using a CP-driven and body-loaded birdcage coil with varying maximum birdcage coil mesh sizes (2 mm, 4 mm, and 6 mm). Bars indicate the radiated power and the power dissipation in the birdcage coil, RF shield, and body load, expressed as percentages of the forward power accepted by the birdcage coil.

**SUPPORTING INFORMATION FIGURE S12:**


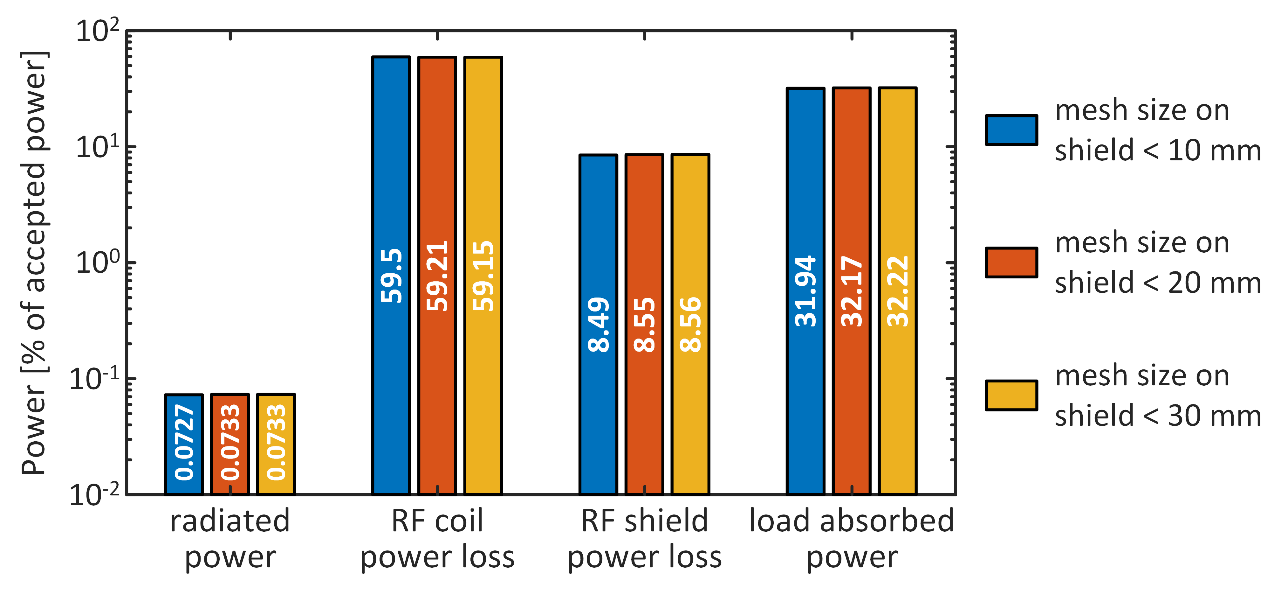


**Figure S12** – Normalized power analysis of an unshielded 1.5T MRI system using a CP-driven, body-loaded birdcage coil with different maximum mesh sizes on the RF shield: 10 mm, 20 mm, and 30 mm. The bars represent the radiated power and the power dissipation within the birdcage coil, RF shield, and body load as percentages of the forward power accepted by the birdcage coil.

**SUPPORTING INFORMATION FIGURE S13:**


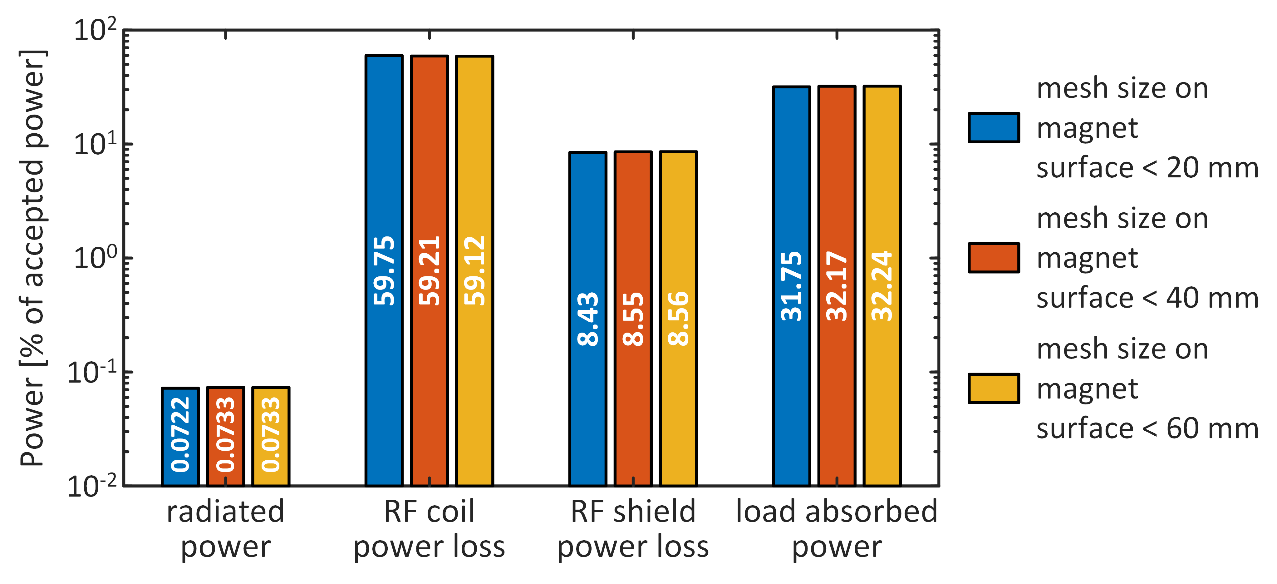


**Figure S13** – Normalized power analysis of an unshielded 1.5T MRI system using a CP-driven birdcage coil loaded with the body for 3 different maximum mesh sizes on the magnet surface (20 mm, 40 mm, and 60 mm). The bars represent the radiated power and the power dissipated in the birdcage coil, RF shield, and body load as percentages of the forward power (power accepted by the birdcage coil).

**SUPPORTING INFORMATION FIGURE S14:**


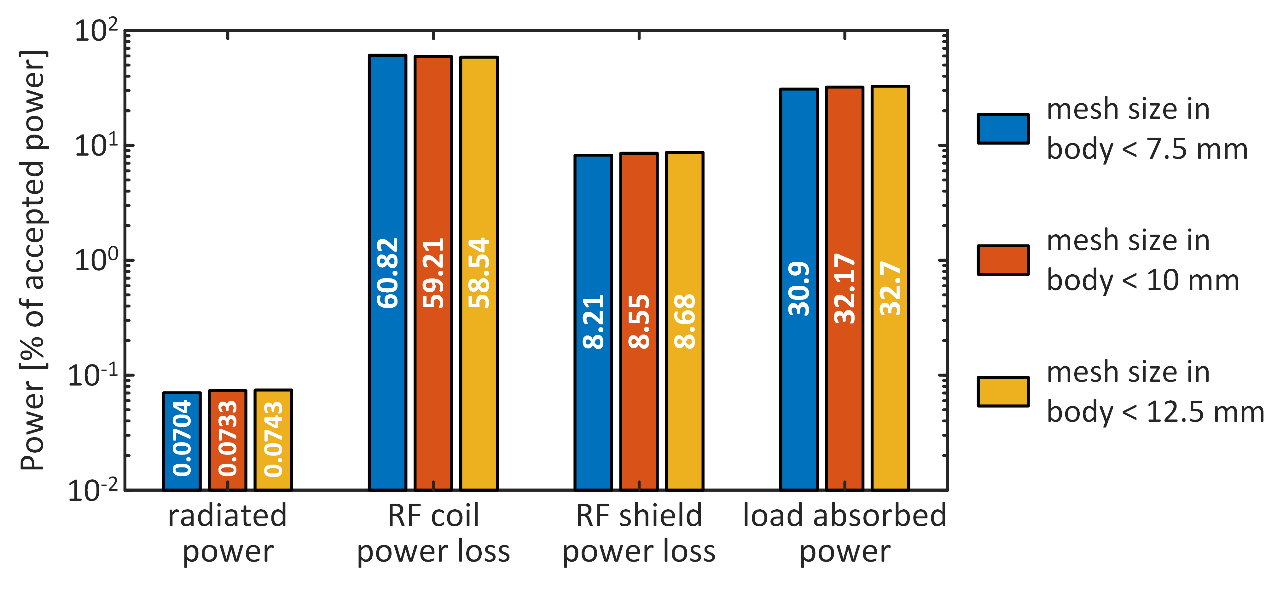


**Figure S14** – Normalized power analysis for an unshielded 1.5T MRI system with a CP-driven birdcage coil loaded with the body model using different maximum mesh sizes within the body model (< 5 mm, < 10 mm, and < 15 mm). The bars show the radiated power and the power dissipation in the birdcage coil, RF shield, and body load expressed as percentages of the forward power (power accepted by the birdcage coil).
